# Mobile Point-of-Care Device Using Molecularly Imprinted Polymer-Based Chemosensors Targeting Interleukin-1β Biomarker

**DOI:** 10.3390/bios13121013

**Published:** 2023-12-05

**Authors:** Rowoon Park, Sangheon Jeon, Jae Won Lee, Jeonghwa Jeong, Young Woo Kwon, Sung Hyun Kim, Joonkyung Jang, Dong-Wook Han, Suck Won Hong

**Affiliations:** 1 Department of Cogno-Mechatronics Engineering, Department of Optics and Mechatronics Engineering, College of Nanoscience and Nanotechnology, Pusan National University, Busan 46241, Republic of Korea; rowoon.p153@gmail.com (R.P.); jsfhse@pusan.ac.kr (S.J.); jik904@pusan.ac.kr (J.W.L.); jh.jeong@pusan.ac.kr (J.J.); nanohan@pusan.ac.kr (D.-W.H.); 2 Department of Optics and Mechatronics Engineering, College of Nanoscience and Nanotechnology, Pusan National University, Busan 46241, Republic of Korea; 3 Engineering Research Center for Color Modulation Extrasensory Cognitive Technology, Pusan National University, Busan 46241, Republic of Korea; handmade4522@gmail.com (Y.W.K.); ksh1332@pusan.ac.kr (S.H.K.); 4 Department of Nanoenergy Engineering, Pusan National University, Busan 46241, Republic of Korea; jkjang@pusan.ac.kr

**Keywords:** molecularly imprinted polymers, interleukin-1β, cytokine, electrochemical impedance spectroscopy, point-of-care testing

## Abstract

Molecularly imprinted polymers (MIPs) have garnered significant attention as a promising material for engineering specific biological receptors with superior chemical complementarity to target molecules. In this study, we present an electrochemical biosensing platform incorporating MIP films for the selective detection of the interleukin-1β (IL-1β) biomarker, particularly suitable for mobile point-of-care testing (POCT) applications. The IL-1β-imprinted biosensors were composed of poly(eriochrome black T (EBT)), including an interlayer of poly(3,4-ethylene dioxythiophene) and a 4-aminothiophenol monolayer, which were electrochemically polymerized simultaneously with template proteins (i.e., IL-1β) on custom flexible screen-printed carbon electrodes (SPCEs). The architecture of the MIP films was designed to enhance the sensor sensitivity and signal stability. This approach involved a straightforward sequential-electropolymerization process and extraction for leaving behind cavities (i.e., rebinding sites), resulting in the efficient production of MIP-based biosensors capable of molecular recognition for selective IL-1β detection. The electrochemical behaviors were comprehensively investigated using cyclic voltammograms and electrochemical impedance spectroscopy responses to assess the imprinting effect on the MIP films formed on the SPCEs. In line with the current trend in in vitro diagnostic medical devices, our simple and effective MIP-based analytical system integrated with mobile POCT devices offers a promising route to the rapid detection of biomarkers, with particular potential for periodontitis screening.

## 1. Introduction

Periodontal disease refers to a disease that occurs in the tooth-supporting tissues rather than the damage of the tooth, and it is mainly caused by the continuous formation of plaque surrounding the tooth [1,2]. When the bacterial film hardens without being removed, it develops into tartar that accumulates and causes the gums to separate from the tooth, creating a gap called a periodontal pocket. If more biofilms grow into the periodontal pocket, the alveolar bone can be destroyed. This chronic periodontitis progression currently represents the leading cause of worldwide inflammatory disease. More importantly, the possible correlations of periodontitis with diabetes and cardiovascular disease have been proven in previous studies for decades [3]. For example, the main causative bacteria of gum disease, called the *red complex*, were detected at high levels in patients with pre-diabetes, and one of the pathogens most frequently extracted from the plaques of patients with carotid atherosclerosis is Porphyromonas gingivalis, the causative agent of periodontitis [4,5,6]. Surprisingly, this pathogen has been also found in brain tissue from Alzheimer’s patients [7]. Despite the quite urgent demand of the diagnostic guidance in the clinical community, the traditional diagnosis of periodontitis mainly involves the bleeding on probing, the probing depth, the tooth mobility, and the use of radiographic examinations to examine the permanent damage of the tooth tissues, which has the disadvantage of making it difficult to accurately judge the degree of the periodontal progression [8].

To assess the subclinical symptoms for the inflammatory response, another current technology used as a standard technique is the enzyme-linked immunosorbent assay (ELISA), which is capable of detecting accurate levels of the biomarkers up to 0.2 pg mL^−1^ via professional operation [9,10]. This technique is sensitive and quantifiable when used with standard antibodies, but it requires time-consuming steps for the analysis process. Disadvantages include the challenges presented by all antibody-based methods, including cross-reactivity and nonspecific signal generation [11]. Therefore, for the diagnosis and monitoring of periodontitis, user-friendly point-of-care testing (POCT) platform technology is continuously being developed based on the electrochemical principles of combining signal transducers by measuring the potential, impedance, capacitance, and electrical conductivity [12,13]. As for in vitro diagnostic (IVD) medical devices, these types of chemosensors with enhanced sensitivity/selectivity and simple operating systems could be the main potential strategy to provide clinicians with valuable information on the condition of the soft-tissue destruction in periodontitis [14]. In this context, versatile approaches for clinical diagnosis methods have been proposed, such as lab-on-chip technology [15,16], lateral-flow assays [17,18], electrochemical electrodes [19,20], surface-enhanced Raman scattering [21,22,23], and integrated semiconducting transistors [24,25]. In view of the biological sensing capability, the creation of recognizable active sites is critically required for the stable physical and chemical interactions in the transducing process to build an advanced POCT system.

Recently, molecularly imprinted polymers (MIPs) have garnered significant attention as the most promising technique to generate customized biological receptors (i.e., target molecules) by providing specified chemical cavities [26,27]. This unique configuration features high compatibility with recognizable biological molecules, such as enzymes, antibodies, and aptamers [28]. In general, the designated MIP structures on the specific surfaces can be produced via the simple polymerization of functional monomers with the cross-linking agent and the target template molecule that has an analogous chemical conformation. Subsequently, the removal of the templates on the surface leaves behind copious cavities, at which the target molecules will go through a complemental rebinding process. MIP technology has been firmly established for several decades and will no doubt be used as a synthetic “*lock-and-key*” to identify targeted analytes with high selectivity. By rapidly developed combinatorial approaches, MIPs have been adopted for a wide range of useful applications, such as biomolecule separation, chemosensors, bioimaging, and drug delivery systems [29,30,31,32,33]. For POCT application, one option is to integrate measurable biomarker indicators based on the MIP materials system for affordable sensitivity and accuracy, extracting molecular evidence on the diagnostic symptoms [34]. 

Here, we present a simple yet robust platform utilizing the MIP technique for protein imprinting to detect a biomarker, interleukin-1β (IL-1β), indicating a diagnosis of periodontitis. Because the abnormal expression of the cytokine levels reflects changes in the physiological environment (i.e., tissue damage), the detection of this specific cytokine offers straightforward precision to monitor the effect of the immune function on the diagnostic symptoms. The intimate integration of the surface-MIP-based biosensing system involved the preparation of three-channel electrodes and a subsequent strategic electropolymerization-designed functional layered thin film for templating the biological recognition sites, so-called “antibody mimics”. To access sufficient chemical selectivity with minimal impedance drift and stability over a wide range of concentrations, we selected eriochrome black T (EBT) as a functional monomer with the IL-1β templates, associated with a highly conductive interlayer of 3,4-ethylene dioxythiophene (EDOT), forming MIP electrodes. The chemically isolated cavities that possess selective recognition capabilities were ready to serve as rebinding sites after the removal of the templates. Based on this biosensor, for the demonstration of an immediate diagnosis, a mobile POCT device was built with a customized relay circuit system to connect the MIP-enabled electrodes with a smartphone for a user-friend interface. By capturing IL-1β on the electrode, the cytokine levels were quantified, and they satisfied the guidelines of periodontitis detection with reliable device-to-device uniformity in the electrochemical performance. In addition, we analyzed the key attributes of the MIP system by evaluating the linear change with varying concentrations of IL-1β in the response with the Nyquist plot using a conventional potentiostat, as well as its selectivity over other interfering biomolecules (e.g., Myo and IgG), within physiologically relevant concentrations. With these technological developments, we believe that, in the near future, the basic principle of our proposed IVD device may be suitable for the high-yield diagnosis of periodontitis on POCT platforms, not only for the early monitoring of the clinical effectiveness of periodontitis treatment, but also to facilitate user-friendly diagnostic tools in long-term-monitoring scenarios.

## 2. Materials and Methods

### 2.1. Materials

Sulfuric acid (H_2_SO_4_) (purity: 98%), 4-Aminothiophenol, (4-AMP) (purity: 96%), EDOT (purity: 97%), EBT, potassium hexacyanoferrate (K_3_[Fe(CN)_6_]), potassium hexacyanoferrate trihydrate (K_4_[Fe(CN)_6_]·3H_2_O), phosphate-buffered saline (PBS) (0.01 M; pH: 7.4), phosphate-buffered solution (PB) (0.1 M, pH: 7.5), sodium hydroxide (NaOH) (purity 99%), and myoglobin (Myo) and immunoglobulin G (IgG) were purchased from Sigma-Aldrich Co. (St. Louis, MO, USA). Interleukin-1β (IL-1β) (MW = 17.4 kDa) was purchased from NKMAX Co. Ltd. (Seongnam, Republic of Korea).

### 2.2. Preparation of Customized SPCEs on Flexible Substrate

The three-channel electrodes were fabricated on flexible polyimide (PI) with a thickness of 100 μm via a homemade screen-printing apparatus consisting of an x-axis stage to position a screen mask, a y-axis stage to control the snap-off distance, a mask tension gauge, a squeegeeing pressure controller, and a vacuum holder. The screen used in this work was a mesh screen stretched on a 320 mm × 320 mm cast aluminum frame (Figure S1). The mesh opening was 200 μm, the wire diameter was ~140 μm, and the open area was 35%. Additionally, the stencil mask was manufactured using a laser-cut method and has a thickness of 50 μm. The carbon working/counter electrodes and Ag/AgCl reference electrodes were defined using a separate meshed screen. After firmly fixing the PI film to the x-axis stage, each electrode was printed in two round trips using carbon ink or Ag/AgCl ink at a snap-off of 0.77 mm while maintaining the tension of the meshed screen mask. By squeezing carbon ink onto the PI film through the meshed screen mask, carbon electrodes were printed with the sizes of the circular working electrode (WE) (diameter: 3 mm), an arc-shaped counter electrode, and the lead lines. The WE and CE transferred onto the PI substrate were thermally cured at 60 °C for 60 min. Similar to the fabrication process of the carbon electrode, an arc-shaped reference electrode was manufactured using Ag/AgCl ink on the PI film and then thermally cured at 60 °C for 60 min. Finally, an adhesive PI tape as an insulating layer was firmly attached to the lead lines of the printed electrodes to define the active area of the electrodes and protect the interconnects from the external environment.

### 2.3. Fabrication of the MIP Sensors

The MIP-based biosensors were fabricated on the customized SPCEs via an electropolymerization method with a potentiostat (CompactStat, Ivium Technologies BV, Eindhoven, the Netherlands). First, the SPCE surface was activated via cyclic voltammetry (CV) cycling under the potential range of −0.65~1.05 V, a scan rate of 50 mV s^−1^, and 10 cycles with a drop of 0.5 M H_2_SO_4_ solution. Next, PEDOT film was electrochemically deposited using 5 mM EDOT in 0.1 M PB onto the activated SPCE surface through chronoamperometry (CA) under optimized conditions (fixed potential: +0.9 V; deposition time: 10 s). Then, PEDOT/SPCEs were incubated using a 2 mM 4-AMP solution (a mixture of EtOH and deionized water in a 5:5 volume ratio) for 1 h at room temperature to promote adhesion between the PEDOT interlayer and PEBT film. After that, the 4-AMP/PEDOT/SPCEs were rinsed away several times with deionized water to eliminate the unreacted 4-AMP molecules and then dried with nitrogen gas. Following the formation of the 4-AMP monolayer on the PEDOT/SPCEs, PEBT thin film templated with IL-1β was electrochemically deposited via CV cycling over the potential range from −0.45 V to +0.9 V at 100 mV s^−1^ for 8 cycles in 0.1 M PB (0.14 × 10^−6^ M IL-1β and 2.5 × 10^−3^ M EBT). The PEBT-based MIP electrodes were washed several times with DI water and dried with nitrogen gas. To form the imprinted cavities in a PEBT-based MIP matrix, the electrodes were immersed in 0.1 M NaOH to remove the IL-1β template under mild magnetic stirring at 200 rpm for 60 min. The nonimprinted polymer (NIP)-based electrodes were prepared using the same method under the absence of the IL-1β template.

### 2.4. Surface Characterization

The surface morphology and topography of the SPCEs for each deposition step were observed using field-emission scanning electron microscopy (FE-SEM) (SUPRA 40 VP, Carl Zeiss SMT, Oberkochen, Germany) and atomic force microscopy (AFM) (XE-100, Park Systems, Suwon, Republic of Korea) in non-contact mode. The XPS spectra of each deposition step were characterized via X-ray photoelectron spectroscopy (XPS) (AXIS Supra, Kratos Analytical Ltd., Manchester, UK) equipped with an Al-Kα excitation source (1486.6 eV).

### 2.5. Characterization of the MIP Sensor Performance

All electrochemical measurements of the MIP- and NIP-based electrodes for the cytokine sensing response were performed using a portable potentiometer (Sensit Smart, PalmSens BV, Netherlands) in 0.01 M PBS (pH: 7.4) containing 5 mM K_4_[Fe(CN)_6_]/K_3_[Fe(CN)_6_] redox probes. CV was conducted in a potential range of −0.3–0.7 V at a 100 mV s^−1^ scan rate and with one sweep cycle. EIS profiles were recorded under the 0.01 Hz–100 kHz frequency range and at 0.1 V potential and 10 mV amplitude. All EIS data were fitted to a Randles equivalent circuit using PSTrace 5.3 software, and the R_ct_ value was derived from the Nyquist plot (frequency response of the electrode/electrolyte system). To evaluate the adsorption behaviors on the IL-1β-imprinted MIP films for the IL-1β target protein, EIS responses were recorded after a 1 h incubation process in IL-1β standard solution (100–1000 pg mL^−1^ in 0.01 M PBS). The specific binding of the IL-1β-imprinted MIP films was investigated in solution samples of IL-1β, Myo, IgG, IL-1β/Myo, and IL-1β/IgG mixed at the same concentration of 300 pg mL^−1^.

### 2.6. Data Analysis and Statistics

Statistical data are expressed and presented as the mean ± SD from three measurements using the MIP sensor chip. Statistical significance was estimated through statistical hypotheses, and the significance of the derived value was assumed to be *p* < 0.05. The reproducibility of the MIP sensor is presented as relative standard deviation. The limit of detection (LOD = (3.3 × SD))/slope of the curve and the limit of quantification (LOQ = (10 × SD))/slope of the curve were derived through correlation with the designed calibration curve. All statistical data were analyzed using Origin-Lab 8.0 software.

### 2.7. Preparation of the Microfluidic Chips

Microfluidic chips were fabricated using the Stereolithography 3D printing technology process. Materials used for 3D printing included stereolithography resin (Clear) from Forms3 (Formlabs, Somerville, MA, USA). The design was 3D-printed onto the support structure at a 45° angle using PreForm software (Formlabs). After printing, the 3D modules were cleaned of residues with isopropanol, dried, placed in a Form Cure (Formlabs) chamber, and exposed to 405 nm UV light at 60 °C for 15 min.

### 2.8. Simulation of Molecular Electrostatic Potential

The electrostatic potential of the pre-polymerization complex to estimate the binding energy in the imprint was calculated. The EBT molecular structure formed with the amino acids, such as alanine, was generated by GaussView 6. The ends of the amino acid chains were hydrogenated to assume a neutral structure. The EBT and amino acid geometry was optimized via the semi-empirical Parametric Method 6 (PM6) with Gaussian 16W software to determine the lengths of the hydrogen bonds, defined as the distance between the electronegative atoms participating in the hydrogen bond [35]. Considering the Mulliken charge of binding oxygen, the EBT molecule was brought close to the amino acid, and stable hydrogen bonds were formed at up to 4 positions. The binding energy of the pre-polymerization complex was calculated via the density functional theory (DFT) method at the B3LYP/6–31+G level with Gaussian 16W software.

## 3. Results and Discussion

### 3.1. Design of MIP-Based Biosensor and Mobile POCT System

**Scheme 1:**
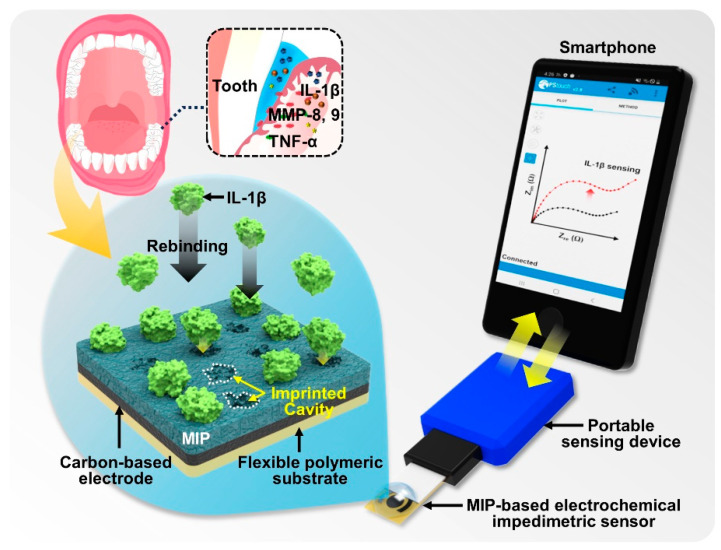
Illustration representing a mobile POCT platform for the scheme of periodontitis screening using MIP-based biosensors detecting IL-1β biomarker.

Scheme 1 displays the conceptual design of an MIP-enabled biosensor featuring a mobile POCT system [36,37]. A specific type of MIP-based biosensor is presented to capture and quantitatively analyze a target molecule found in the oral cavity. In our experimental design, as previously mentioned, we prepared MIP-loaded electrodes by mixing functional monomers with the target template molecule (i.e., the IL-1β cytokine), and then electropolymerization on a highly conductive electrode surface was utilized for a sequential deposition of EDOT/EBT onto the SPE surface [38]. As a fundamental approach, the choice of IL-1β, among other proinflammatory cytokines (target template), such as tumor necrosis factor (TNF)-α and IL-6, is important because it has been recognized in periodontal inflammation and tissue damage [39]. Thus, IL-1β stands out as a potent stimulator of periodontal tissue destruction due to its role in enhancing the expressions of collagenases and matrix metalloproteinases (MMPs), which, in turn, lead to extracellular matrix degradation, bone resorption, and tissue destruction [40]. As reported previously [41], elevated levels of IL-1β are frequently detected in the saliva and gingival crevicular fluid (GCF) of periodontitis patients, compared to healthy controls. For a practical application, the MIP-based biosensor detecting IL-1β holds promise beyond periodontitis, as the inflammatory cytokine IL-1β is also associated with systemic inflammatory diseases such as gout, osteoarthritis, vascular disease, heart failure, or stroke [42,43].

As reported previously in terms of the technique, delicate electropolymerization to produce MIPs has been acknowledged for its suitability to crafting highly sensitive sensing properties in the categorized surface-MIP technology [44,45]. It exhibits significant promise for enhancing sensitivity, especially for small biomolecules, such as proteins. Further, because the MIP technique is highly dependent on the imprinting strategy to generate an “artificial antibody”-integrated polymeric active layer, finely tuned parameters (e.g., scan rate, cycle numbers, and potential window) for electropolymerization on the electrode surface are critically important in controlling the stacking of the monomer layers. In this study, control experiments on the electropolymerization of EDOT and EBT were guided by previous reports [46,47]. Under the controlled condition, the boundaries between the imprinted templates and adjacent polymer matrix can be defined by the shape of the complemental recognition sites according to the size of the molecules. Following this electropolymerization process, the template molecule is ready to be removed from the polymer matrix on the SPE surface, leaving behind target-imprinted cavities characterized by a target molecule that is complementary in size, molecular shape, functional end group, and molecular orientation [48]. After the appropriate removal of the imprinted templates, the copious cavities on the surface of the polymer matrix serve as artificial antibodies that mimic IL-1β, facilitating selective target rebinding. A key element of the operation of MIP-based biosensors is the accurate rebinding of the target molecules (i.e., IL-1β) on the active electrode to transduce the signal changes, such as impedance. Thus, a pivotal aspect of the molecular design can be attributed to the rational synthetic route for the deposition layer of monomers with an appropriate number of templates [49]; we discuss this in later sections. Indeed, by integrating additional redox probes into surface-MIP-based biosensing systems, the extent of the molecular recognition in this recombination can be translated into typical electrochemical signals. As a user-friendly toolbox, our MIP-based biosensing system leverages smartphone functions for the measurement and transmission of electrochemical signals (e.g., impedance). In other words, it includes the capability to transmit measured results using a portable electrochemical potentiometer (lower-right image in Scheme 1). The mobile POCT system presented here may contribute to progress in the analysis of dynamic interactions on active electrodes, detecting biomarkers within biological fluids. As a customizable monitoring kit that easily maintains stability, MIP-based biomolecular detection technology may not only advance measurement methodologies but also improve the user accessibility of mobile systems [50].

### 3.2. Production of Customized SPCEs on Flexible Substrate

**Figure 1:**
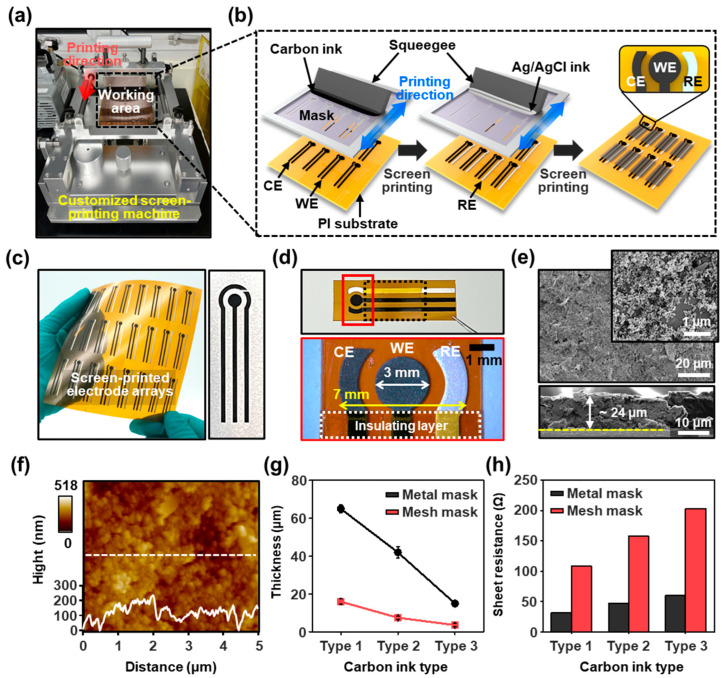
(**a**) Photograph of the experimental setup of a screen-printing apparatus for printing carbon and silver-based electrodes onto a target PI substrate. (**b**) Schematic illustration of the sequential-printing process to prepare the array of SPCEs. (**c**) Digital images of the array of SPCEs manufactured on the PI substrate by the sequential-printing process (left) and the enlarged image of an individual electrode (metal mask, right). (**d**) Digital images of a single SPCE (upper) and the magnified area of the WE, CE, and RE (lower), as a result of the printing method. (**e**) SEM images representing surface morphology of carbon electrode with graphite flake and carbon particles. (**f**) AFM image showing the 2D height profile and surface roughness of the working electrode; the dotted white line corresponds to the line-scanned location. Comparison of the (**g**) thickness level and (**h**) electrical conductivity of the carbon layer manufactured by the type of conductive ink using the mesh/metal masks.

**Figure 2:**
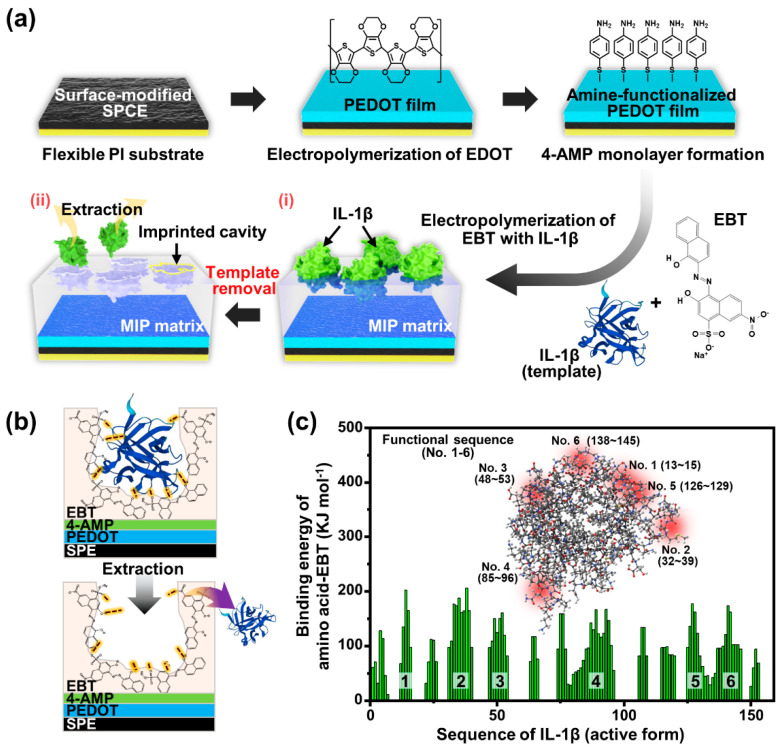
(**a**) Stepwise illustration of the electropolymerization of MIP-based biosensor for IL-1β cytokine detection, including the pretreatment of SPCEs, PEDOT deposition (interlayer), the formation of a 4-AMP self-assembled monolayer (linker), and PEBT electropolymerization with IL-1β protein (MIP film, (**i**)); after the extraction of the templates, molecularly imprinted cavities can be formed (**ii**). (**b**) Detailed schematic diagram for the formation of molecularly imprinted cavities complementary in size and shape to the template after the extraction of the IL-1β template. (**c**) The binding energy of amino acid/EBT according to the sequence of IL-1β, based on molecular electrostatic potential calculations of the EBT/amino acid molecules. Some sequences are inside the IL-1β (e.g., sequences 55–62) and have regions that do not contribute to the binding energy, which was determined through the X-ray diffraction results (PDB: 1HIB).

Prior to the electrochemical polymerization of the MIPs, we developed a process to produce tailored SPEs for the compatible assessment of the synthesis/measurement equipment and POCT systems. In this approach, a small-scale screen-printing setup was used to prepare three-channel electrodes on a provided substrate, as shown in Figure 1a. The homemade printing apparatus consists of an x-axis stage to position a screen mask, a y-axis stage to control the snap-off distance, a mask tension gauge, a squeegeeing pressure controller, and a vacuum holder to secure the flat fixation of the provided substrate. We first selected a flexible substrate (polyimide (PI)), considering the expandability for POCT applications with microfluidic-channel-combined MIP-based sensing platforms for detecting IL-1β [51]. Figure 1b schematically illustrates the manufacturing process of a three-channel electrode designed with the working, counter, and reference regions. First, carbon ink was applied to the screen mask (opening region: mesh numbers of 200 counts per inch) for the working and counter electrodes (left panel in Figure 1b), and then Ag/AgCl ink for the reference electrode was squeezed onto the carbon ink-printed substrate with another screen mask (middle panel in Figure 1b). At this stage, the alignment of the screen masks is an important parameter in addition to the viscosity and squeezing speed of each ink. To print the conductive inks onto the provided substrate (typical size: 320 × 320 mm^2^), we set optimized conditions by passing a squeegee with constant pressure through the open areas in the screen mask. Once the experimental conditions are set, this simple technique is highly effective for transferring the conductive inks onto the receiving substrate to create a patterned electrode array of the desired configuration. This process resulted in individual sets of three-channel electrode arrays with a circular working electrode (WE) (diameter: 3 mm) in the center, surrounded by arc-shaped counter/reference electrodes (CE/RE) (width: 1 mm). The substrate was thermally cured at 60 °C for 60 min to fully evaporate the residual solvent and induce dense films. Finally, an insulating layer (i.e., polyimide tape) was attached to the lead lines of the printed electrodes, except for the active electrodes and connection terminals (right panel in Figure 1b). As presented in Figure 1c, an array of customized SPCEs was produced via this technique (48 each at a time) on a flexible PI substrate. The magnified optical micrograph on the patterned electrode surface shows a clear contrast on the PI substrate, indicating 1:1 pattern generation with carbon and Ag/AgCl ink (Figure 1d). To observe the detailed morphology of the electrode surface, we used SEM, as appears in Figure 1e and Figure S2. In the typical structure of the SPCEs, densely packed large grains of graphite flakes (~1 μm) and carbon particles (~25 nm) were well distributed over the entire surface area, with a thickness range of ~24 μm, where the relatively flat height profile indicates the controlled uniform film. Further AFM analysis was also performed to investigate the surface roughness of the carbon electrode, as shown in Figure 2f. The average surface roughness (*R*_a_) of the screen-printed working surface was ~20.8 nm, and the root-mean-square (RMS) deviation in the surface roughness (*R*_q_) was ~26.9 nm. As measured, the surfaces of the printed electrodes (Figure 1e,f) indicate slightly rough topological features with some porosity. This characteristic microstructure of the screen-printed area can be attributed to the insufficient level of solidification in low-temperature diffusion by the use of a volatile organic solvent during the drying process [52]. 

After setting the film formation process, we optimized the thickness range and the electrical conductivity by applying different types of masks and carbon inks. Because the quality of the SPCEs could be determined by the types of conductive ink and the opening area of the mask, a survey of the correlation between the conductive inks and their trends of deposition through the exposed regions of the patterned mask can be an important factor [53]. In other words, when the squeezed inks adhere to the PI substrate, subtle physical and chemical interactions, as the solvent evaporates, may produce large deviations in the solid-phase separation or aggregation of the carbon particles [54]. Based on earlier works, we additionally considered a metal stencil mask (i.e., without mesh), in addition to the typically used mesh screen mask, to utilize three commercially available carbon inks defined by graphite flakes and carbon particle ratios. We postulated that there may be a trade-off between the penetrating rate of the carbon inks with fixed viscosity and the film formation, depending on the ratio of microscale graphite flakes and nanoscale carbon particles. As reported previously [55], the parameter decision on the mesh type or metal stencil mask was primarily attributed to the open area and the initial ink properties. As presented in Table S1, each carbon ink was classified by the ratio of graphite flakes/carbon particles and the corresponding specific viscosity. As shown in the control experiments (Figure 1e), the graphite flakes were measured as layered planar structures of a few microscales, while the carbon particles were spherical and much smaller in shape [56]. When passing through the opening area of each mask during the printing process on a provided substrate, the graphite flakes and carbon particles mixed with the binder resin can be spontaneously aggregated and compressed with a certain pressure to form a film structure. Therefore, heterostructured films containing mixed materials naturally form porous surfaces with higher surface areas [57]. Because the composition ratio of graphite flakes and carbon particles affects the film thickness and surface morphology in the SPCE structure, the related electrical properties are critically important for the final sensing performance. The graphite flakes are highly conductive due to their layered structure and delocalized electrons, which are the main materials for electrical conduction. Thus, increasing the ratio of graphite flakes in the electrode composition can obviously enhance its electrical conductivity, enabling better electron transfer during electrochemical reactions, although the inclusion of carbon particles may slightly reduce the electrical conductivity compared to an electrode consisting of graphite flakes alone. However, the higher content of carbon particles can assist in forming a higher surface area of the printed electrode, which also improves the reactivity due to inherent defects or functional groups on their surfaces and is more favorable for electrochemical sensing applications. Depending on the applied conductive inks, it is necessary to set an optimized process with a composition ratio of graphite flakes and carbon particles to produce SPCEs. In our experimental approach, maintaining the stability of the electrode over time is desirable by improving the electrical conductivity of the electrode for interaction with electrolytes or the attachment of electroactive species [58]. As tested in Figure 1g, the experimental results show a relationship between the perforated conditions of each mask and the thickness range of the printed carbon electrodes for different types of carbon inks. It was found that the range of the film thickness of the carbon electrodes was clearly related to the provided carbon ink’s viscosity and simultaneously open areas. In a comparison of the two different masks, the more permeable mask to the inks (i.e., metal stencil mask) was preferred to form the electrode array within linearly reduced thickness levels. In contrast, in the case of the mesh screen mask, although a linear relationship of the carbon ink’s viscosity with small differences in the film thickness was yielded, the lower viscosity of the carbon ink was less effective for electrode formation (i.e., high resistance for SPCEs). This summarized result can be attributed to the effect of the meshes on the screen, as large microscale graphite flakes were filtered when passing through the meshes. The film thickness range of the carbon electrodes according to the ink type and mask was directly reflected in the conductivity, as shown in Figure 1h, where the carbon electrodes fabricated with a high graphite flake ratio displayed lower sheet resistance, regardless of the mesh type; the electrical conductivity was increased in the case of a mesh screen mask with increased graphite content. However, within our experimental condition, the carbon electrode produced from the metal stencil mask (i.e., more openings) did not lead to large variations in the conductivity, independent of the ink type. Although the free flow of carbon inks was observed in the open area in the metal stencil mask, the selected ink (i.e., type 3 carbon ink) resulted in affordable resistance ranges. Based on these results, we established parametric conditions for the mass generation of SPCEs, which serve as the basis for fabricating MIP electrodes for electrochemical modification.

### 3.3. MIP Formation on SPCEs Using Electropolymerization

As schematically illustrated in Figure 2a, we performed the electropolymerization process on the SPCEs to prepare MIP-based biosensors, especially for IL-1β detection. The sequential processes involved the pretreatment of the SPCE surface, the electrochemical deposition of the PEDOT interlayer, the 4-AMP self-assembled monolayer application, PEBT-based MIP electropolymerization, and the final template extraction from the MIP matrix. At the initial stage of the MIP film formation, the surface of the active carbon electrode region (i.e., WE) was electrochemically oxidized via consecutive CV cycles in 0.5 M of H_2_SO_4_ solution. This procedure is required to eliminate the impurities from the electrode surface and electrochemically activate the WE. As previously reported, an electrochemical pretreatment is greatly effective in purifying the WE’s surface by oxidizing and expulsing the residual contaminants (e.g., organic binders) surrounded at the surfaces of the carbon particles [59]. After the electrochemical activation procedure, the electron transfer capacity of the WE reached saturation and remained highly reproducible throughout the manufactured SPCEs (Figure S3). Subsequently, the PEDOT thin film was electropolymerized on an electrochemically activated WE surface under a fixed potential (10 s at 0.9 V) using the CA technique to enhance the electrical response of the MIP-based electrode. In this step, the reason for applying the CA technique is that the current supplied to the electrodes can be precisely quantified, which facilitates the control of the film thickness [60]. When the constant potentials are subjected to surface-activated carbon electrodes (i.e., WEs), EDOT monomers are rapidly oxidized to highly reactive radical cations by electron transfer, and then the radical cations are condensed to dimers, trimers, and longer oligomers [61]. These EDOT oligomers nucleate on the WE surface and precipitate with an increasing chain length, resulting in insoluble PEDOT chains, comprising from ~6 to 20 EDOT monomers [62]. Thus, these EDOT oligomers merge to form a continuous PEDOT film at the WE, which leads to inward growth from the edge of the electrode with the highest charge density [63]. Compared to surface-activated SPCEs (Figure S3), the Nyquist plot of PEDOT-coated SPCEs exhibited a semicircular pattern, implying their improved electrical conductivity (Figure S4). It is worth noting that the electrochemically deposited PEDOT film on the WE surface reduces in impedance and further compensates for the increased impedance following the sequential process for MIP polymerization. Next, a 4-AMP monolayer was self-assembled on the PEDOT/SPCE to promote adhesion between the monomer units (i.e., EBT) and the PEDOT film. The PEDOT film was incubated in a 4-AMP solution for 1 h, where the 4-AMP layer served as a linker connecting the PEDOT with the subsequent PEBT film [64]. Favorable interaction of the thiol group in the 4-AMP with EDOT molecules on the PEDOT film means that it is ready to form disulfide bridges, and the amine-aromatic ring covalently bonds with the MIP film (i.e., PEBT). We expected that this chemical configuration of the stacked layers (i.e., 4-AMP/PEDOT/SPCE) would effectively assist the tight binding of the PEBT film without the degradation of the sensing performance (Figure S5). Following the 4-AMP monolayer formation, the PEBT-based MIP film was electrochemically deposited via CV cycling over a range of from −0.45 to +0.9 V at 100 mV s^−1^ for 10 cycles in PBS buffer (0.14 × 10^−6^ M IL-1β and 2.5 × 10^−3^ M EBT). 

We selected the EBT monomer as the MIP material to template the IL-1β protein in the imprinting matrix, considering the abundance of functional groups in the chemical structure, such as the nitro, sulfonic acid, and hydroxyl groups. Figure S6 shows the CV curves during the electrodeposition of EBT on the 4-AMP/PEDOT/SPCEs with and without IL-1β templates. Based on the collected data, two representative anodic peaks during electropolymerization were found. The irreversible oxidation peaks in the first cycle were confirmed at the potential windows of +0.15 V and +0.46 V. As observed previously [46], the first peak corresponds to the oxidation process of the phenolic hydroxyl group to the benzoquinone, and the second peak represents the azo bond to the diimine structure. The gradual decrease in the oxidation peak with the increasing cycle number indicates that the PEBT film was successfully deposited onto the 4-AMP/PEDOT/SPCE surface, suggesting that the electrochemically grown polymer film maintained its nonconductive characteristics. Additionally, the IL-1β-imprinted EBT films exhibited lower peak currents in the first cycle compared to the nonimprinted EBT films due to the interaction of the IL-1β with EBT monomers and the incorporation of IL-1β insulators into the polymer structure. The current values for both oxidation peaks decreased linearly with increasing cycles up to 10 scans; after that, the change in the current was negligible. By the above sequential process, the MIP matrix consisting of PEBT templated with IL-1β was prepared on the SPCE surface (Figure 2a(i)). Finally, the removal of the templates (i.e., IL-1β) in the PEBT matrix was conducted to generate cavities on the MIP surface by immersing the samples in 0.1 M of NaOH solution for 1 h (Figure 2a(ii)); at this stage, the NIP samples (i.e., without IL-1β templates) were also applied to the same solution to impose the identical surface condition. Figure 2b schematically illustrates the extraction process of the IL-1β template entrapping the surface of the PEBT film to form the imprinted cavities with the chemical rebinding ability for the IL-1β protein. As noted earlier, during electropolymerization, EBT monomers can easily bind the side chains of IL-1β (i.e., glutamine (Gln), asparagine (Asn), serine (Ser), and thereonin (Thr)) by strong hydrogen bonds, including the additional assistance of electron-rich oxygen and the negative-charged function group –SO_3_ [65]. Moreover, the aromatic ring in the PEBT structure enables the support of the π–π interaction with IL-1β [66]. As the alkaline solution extraction progressed, the non-covalent bonding between the PEBT matrix and the IL-1β was dissociated, forming the imprinted cavities that are complementary both sterically and chemically to the template after the complete removal of IL-1β proteins (lower panel in Figure 2b). Consequently, a “*molecular memory*” sensor can be generated by the surface-imprinted PEBT film that allows for the capture of the IL-1β with a high affinity when exposed to IL-1β-containing analytes. 

### 3.4. Optimization of Interlayer-Supported MIP Electrodes

The optimized electropolymerization conditions for each functional layer enabling the MIP electrodes proposed in this study stand as critical determinants influencing the molecular sensing ability to recognize IL-1β and the overall reproducibility. Consequently, various parameters were systemically explored for the MIP assembly, including interlayers. Primarily, we deposited the highly conductive PEDOT film onto the SPCE surface to enhance the sensitivity of the assay response, as denoted by the relative *R*_ct_ values between successive concentrations. As a fundamental technique, EIS analysis is an easy approach to characterizing the surface-modified electrode [67], in which the semicircular portion observed at higher frequencies indicates a process with limited electron transfer, while the linear portion at lower frequencies is related to diffusion. Hence, the diameter of the semicircle corresponds to the charge transfer resistance (*R*_ct_), and this parameter is influenced by the dielectric and insulating properties at the electrode/electrolyte interface [68]. Based on this principle, the PEDOT was strategically integrated as an interlayer, serving as a conduit between the carbon electrode and the PEBT film, in which this inclusion of an intermediate component in the MIP structure aimed to augment the electrical properties of the MIP biosensor [69]. As depicted in Figure S7, the optimization process encompassed determining the conductivity for the PEDOT film. To achieve this, we monitored the CV characteristics of the PEDOT/SPCE, which was fabricated using CA with adjustments in the deposition voltages and times, all within a 0.1 M PBS buffer containing 5 mM of EDOT. When we held the deposition time constant at 10 s across different voltage ranges, it was observed that the PEDOT films that electropolymerized at 0.9 V exhibited the highest peak current responses, compared to the other potential widows (Figure S7a). Moreover, at the deposition time of electropolymerization at a fixed voltage of 0.9 V (i.e., 10 s), the highest peak current value of the PEDOT film was clearly seen in the slightly adjusted time ranges (Figure S7b). Given that a high peak current value in the CV graph signifies a robust electron transfer capability, we adopted these voltage (0.9 V) and time (10 s) conditions for PEDOT electropolymerization. These conditions were expected to provide a consistent electrical performance and maintain the reproducibility of the PEDOT/SPCE (Figure S4) under our experimental conditions.

Importantly, we next systematically optimized the monomer/template concentration ratios, number of polymerization cycles, and recombination time to tune the sensitivity of the PEBT-based MIP sensor (Figure S8). In practical applications, the provided cavities serve as electrolyte/electrode interfaces through which redox probes can reach. Therefore, the molecularly imprinted layer should be ensured in an optimized chemical state to allow for the efficient electron transfer of surface-binding events to the underlying WE [31]. In general, the sensitivity of the MIP-based biosensors can be determined by the quality of the imprinted cavities on the electrode’s surface. Moreover, associated with the thickness of the MIP film [70], there is a subtle correlation between the thickness parameter and the reliability of the biosensor. For example, although the cavity numbers increase with the increase in the thickness of the imprinted film, thicker imprinted films may lead to the slow diffusion of the target analytes to the imprinted cavities and inefficient rebinding interactions on the MIP electrode [71]. In other words, MIP film that is too thin may be insufficient for recognizing the IL-1β target protein, and the creation of a thick film may result in the partial entrapment of templates within the electropolymerized PEBT matrix (Figure S8a) [72]. Thus, to determine the optimized thickness of MIP film, we explored the impedimetric response of each sample fabricated as a function of the number of CV cycles (2, 4, 6, 8, and 10 scans), as plotted in Figure S8b. In our materials system, the *R*_ct_ values increased as the number of electropolymerization cycles increased, which was due to the hindrance of the electron transfer to the electrode interface depending on the MIP film thickness. When exceeding 10 cycles of electropolymerization, each MIP-based electrode presented saturated impedance, a condition in which a dense MIP film with a thickness over the electron transport range can be assumed. In this state, when the electron transport between the electrode and the monomer begins to decrease, the continuous electropolymerization can be blocked by preventing the monomer from precipitating onto the electrode surface. Therefore, based on EIS data, the MIP film thickness was adjusted to 10 scanning cycles for the PEBT electropolymerization, which is expected to provide the most sensitive detection performance of MIP-based sensors. 

In addition to the findings presented above, it is crucial to consider the significant influence of the monomer/template ratio as one of the pivotal parameters that profoundly impacts both the quantity and the configuration of the imprinted cavities within the MIP matrix. Thus, the precise adjustment of this parameter must be optimized to ensure the performance of the MIP sensors. To look into the effects of the monomer concentration on the electrochemical responses of the IL-1β-imprinted MIP electrodes, we crafted the MIP films using a constant IL-1β concentration (i.e., 0.14 × 10^−6^ M) and control solutions with varying concentrations of EBT monomers, ranging from 1.0 to 7.5 mM. Subsequent to the extraction of IL-1β, the IL-1β-imprinted MIP electrodes were characterized using EIS in PBS solution (pH = 7.4) containing 5 mM of [Fe(CN)_6_]^3−/4−^ electrolytes after 30 min of incubation in the presence of IL-1β (100 pg mL^−1^). Figure S8c shows the normalized Δ*R*_ct_/*R*_ct(Blank)_ as a function of the monomer concentration (i.e., *R*_ct_ − *R*_ct(Blank)_ = Δ*R*_ct_), derived from the Nyquist plot of the EIS response. This parameter serves as a key indicator of the sensitivity of the MIP-based sensor, reflecting the change in the *R*_ct_ value due to the adsorption of IL-1β into the imprinted cavities relative to the *R*_ct(Blank)_ value, which is obtained after the template extraction process. Figure S8c conveys that the maximum value of the Δ*R*_ct_/*R*_ct(Blank)_ was attained at a monomer concentration of 2.5 mM. Beyond this threshold, with the increasing monomer concentration, the Δ*R*_ct_/*R*_ct(Blank)_ values exhibited a gradual reduction. This behavior can be attributed to the formation of an excessively thick MIP layer at higher monomer concentrations. Consequently, the template embedded within this MIP matrix becomes challenging to remove, impeding the creation of voids and, in turn, reducing the sensor’s detection capacity. This is consistent with previous validation experiments showing that thicker films have fewer surface-imprinted cavities. Consequently, a monomer concentration of 2.5 mM for EBT was considered optimal for further investigations.

At this stage, prior to optimizing the formation of MIP films, a preliminary simulation study was also conducted to assess the binding forces between the IL-1β protein and EBP monomer using the DFT method [35]. As shown in Figure S9, the docking structures driven by the electrostatic interaction forces of the amino acid (i.e., the partial structure of the IL-1β template molecule) and EBT monomer were quantitatively estimated. This numerical examination holds significant importance in our study because MIP selectivity, driven by non-covalent interactions, is fundamentally influenced by the nature of the interaction between the target analyte and functional monomers during the polymerization process, particularly in terms of the specific bond strength [73]. To compare the interaction forces at the final polymerization step, we calculated the molecular electrostatic potential for each EBT and amino acid unit sequence and for the combined complex. The binding energy of the pre-polymerization complex was approximated from the following equation according to the polarizable continuum model [74]:

Binding energy (Δ*E*) = *E*_complex_ − *E*_EBT_ − *E*_amino acid_ (1)

where *E* is the electrostatic surface potential analyzed via DFT simulation. The calculation was performed for a partial sum, excluding interactions beyond neighboring EBT/amino acids (for detailed calculations, please refer to Supplementary Table S2). As demonstrated in our simulation results in Figure S9, EBT can form non-covalent bonds with four or more amino acid sequences simultaneously. We then averaged the binding energies for the nearest amino acids, as depicted in Figure 2c. Consequently, several representative functional sequences were identified in the six positions, such as SQQ (sequences 13–15), QGQDMEQQV (sequences 32–39), QGEESN (sequences 48–53), DPKNYPKKKM (sequences 86–95), QAEN (sequences 126–129), and KGGQDITD (sequences 138–145). It is widely recognized that the proportion of complex formation before polymerization correlates with the number of highly selective recognition sites in MIP systems because only about 1% of the theoretical binding sites yield moderate-to-high affinities [75]. This implies that the probability of a protein binding to an imprinted site is greatly reduced when other proteins with different sequences are docked. Some EBT molecules that diffuse into the electrostatically neutral region do not exhibit specific binding to amino acids and may interact competitively with the solvent (i.e., water) [76], which may not significantly contribute to the binding energy of the MIP imprinting after polymerization. Instead, they may participate in the cross-linking of the PEBT matrix or form chemical bonds with 4-AMP. In contrast, the functional sequence region represents the binding specificity to the aforementioned amino acid sequences in the MIP imprint, determining its selectivity concerning other proteins. In the inset in Figure 2c, the 3D structure of the active form of IL-1β is visualized [77], where the locations of the functional sequences are marked with red spots. These active regions act as non-covalent bonds that bind PEBT to IL-1β (see also Figure 2b). As shown in Table S2, the protein sequence of IL-1β showed the largest variation in the three amino acid regions (i.e., aspartic acid, asparagine, and glutamine), with 58 active sites for EBT per cytokine protein of IL-1β (Table S3). Considering the molar masses of EBT (461.38 g mol^−1^) and IL-1β (active form of 153 sequences; total structure weight: 17.35 kDa), it is expected from the simulation results that all the EBT molecules will be positioned around IL-1β in an ideal equilibrium state when mixed at 2180:1 in the pre-polymerization stage. Based on these results, MIP electropolymerization experiments were performed by adjusting the ratio of EBT to IL-1β from 2000 up to 20,000. On the one hand, fully mixed EBT can be expected to strengthen the polymeric matrix of the MIP film, contributing to the structural stability before/after extraction [74,76]. On the other hand, as the amount of EBT mixing increases, the density of the imprint may decrease, making the sensor less sensitive.

Lastly, the recombination time of IL-1β was explored on the surfaces of the template-extracted MIP films. After the IL-1β was introduced onto the MIP electrodes for durations of from 1 to 60 min with varying concentrations of 100, 300, and 500 pg mL^−1^, EIS was employed using a PBS solution (pH 7.4) with 5 mM of [Fe(CN)6]^3−/4−^. As illustrated in Figure S10, the normalized values of the Δ*R*_ct_/*R*_ct(Blank)_ were plotted as a function of the adsorption time to compare the relative changes within the applied concentration ranges. As the IL-1β adsorbed into the imprinted cavities in the MIP matrix, it impeded the diffusion of the redox probe, resulting in increased Δ*R*_ct_ values. Notably, changes in the Δ*R*_ct_/*R*_ct(Blank)_ were readily identified in proportion to the quantity of IL-1β present in the standard solution during the same adsorption period. During the incubation with IL-1β concentrations of 100 and 300 pg mL^−1^, the Δ*R*_ct_/*R*_ct(Blank)_ values reached saturation after 10 min. In comparison, a steady state was observed within 30–45 min at a higher concentration level (i.e., 500 pg mL^−1^). Therefore, in our measurement system, the recombination time was set as 45 min for the optimal duration for IL-1β detection on the surface of the PEBT-based MIP biosensor. 

### 3.5. Surface Characterization of PEBT-Based MIP Films

**Figure 3:**
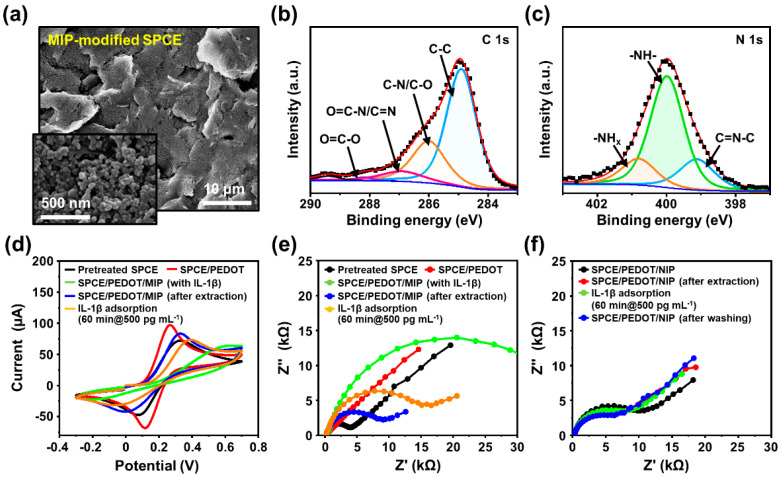
(**a**) SEM images representing the surface morphology of the MIP film after extraction of the IL-1β template. (**b**,**c**) High-resolution XPS survey spectra for MIP-modified electrode after the sequential-electropolymerization steps. (**d**) Cyclic voltammograms recorded in 0.01 M PBS containing 5 mM [Fe(CN)_6_]^3−/4−^ redox probe with a scan rate of 100 mV s^−1^ for the stepwise-modified surfaces of SPCEs. EIS response for stepwise-modified surfaces of (**e**) MIP- and (**f**) NIP-modified electrodes; Nyquist plots were recorded at the amplitude of 10 mV and the frequency ranged from 0.01 to 100 kHz with 0.1 V potential in 0.01 M PBS containing 5 mM [Fe(CN)_6_]^3−/4−^.

Figure 3a and Figure S11 show SEM images of the IL-1β-imprinted and nonimprinted EBT films on the prepared electrodes (i.e., 4-AMP/PEDOT/SPCEs). The IL-1β-templated PEBT film displayed a uniform surface morphology, retaining the appearance of SPCEs in the form of densely packed large grains of graphite flakes and carbon particles. The electropolymerized MIP films were formed along the coarse topological features of the SPCEs with high porosities and increased surface areas, which can promote IL-1β diffusion into the imprinted cavities exposed to the characteristic surface structure during the recombination process, thereby improving the sensitivity of MIP-based biosensors [78]. In addition, the SEM images in Figure S11 show that the nonimprinted PEBT film produced by the same electropolymerization has a similar morphological structure to the IL-1β-templated PEBT film. The topological features of the MIP film before/after IL-1β extraction were examined to approximately estimate the height profile and surface roughness (Figure S12). Consistent with the SEM images, the surface morphology in the AFM images was similar, and only the surface roughness showed a difference, with RMS values of 29.9 ± 5.7 nm before the IL-1β extraction and 42.6 ± 4.4 nm for the completely extracted MIP films. As measured, the IL-1β-imprinted MIP films represented a roughness of more than ~10 nm compared to the NIP films. These differences were attributed to the rigorous extraction of the IL-1β templates from the PEBT film after being flattened by the polymerization process; these results of the AFM characterization agree with previous reports on biomolecule imprinting [38]. Thus, the observation of the surface roughness of the extracted film could be an indirect evaluation of the surface-MIP film.

In addition to the surface morphological evolution of the MIP films at each imprinting/extraction step, the chemical bonding state between the monomer and template must be explored to ensure successful imprinting in the polymer matrix. For the molecular imprinting of proteins as templates for MIPs, it is difficult to identify the structural conformation of the residual proteins in the interpenetrated polymer networks because of the relatively small size of the biomolecules. However, XPS measurements might extract some clues, and some clues might be extracted from the monomer–template complex. As presented in Figure 3b,c, and Figure S13, the chemical compositions of the MIP films were finely evaluated to confirm the formation of the MIPs after the sequential-electropolymerization steps, as summarized in Table S4, which presents the XPS-derived surface atomic concentration ratios at each layer-deposition stage. In the high-resolution XPS spectra for the IL-1β-templated PEBT film on the 4-AMP/PEDOT/SPCE, the strong C 1s peaks detected in the MIP film after the IL-1β extraction indicate four major components, which are attributed to different carbon atoms within the polymer matrix (Figure 3b and Figure S13a). Specifically, as shown in Figure 2d, each component in the chemical bonding group separately reveals O=C–O (288.2 eV), O=C–N/C=N (287 eV), C–N/C–O (286.5 eV), and C–C (284.8 eV) [79,80]. In contrast, the C 1s spectra of the MIP/4-AM/PEDOT/SPCE represent similar chemical features of the individual components compared with the PEDOT/SPCE and 4-AMP/PEDOT/SPCE (Figure S13a). Within this chemical-group similarity, we next considered the N 1s component as a unique elemental marker frequently found in the structure of the protein template that could improve the IL-1β imprinting in the polymer matrix. The N 1s peak was monitored in the XPS spectra at each step produced via the sequential-polymerization process. For example, the N 1s component was not detected in the case of the electrodeposited PEDOT film, and the characteristic peaks of NH_2_ (400.4 eV), NH (399.9 eV), and C=N–C (399.2 eV) [80] were observed after 4-AMP formation and MIP electropolymerization (Figure S13b). As the 4-AMP was assembled on the PEDOT/SPCE, a strong –NH_2_ bond appeared due to the complemental binding from the –SH group in the 4-AMP, which also contains an amine group in the aromatic and thiophene ring of PEDOT. Furthermore, after the electropolymerization of the PEBT films with the IL-1β templates, it was found that the NH_2_ bonds were reduced via hydrogen bonding with hydroxyl groups of EBT, while the intensity of the NH increased (Figure 3c). Additionally, the atomic content of nitrogen on the MIP surface containing IL-1β in the PEBT film was found to be increased by ~50% compared to the surface state of the 4-AMP/PEDOT/SPCE. Although the –NH peak at 399.9 eV was not observed as a distinct peak for the peptide bond (O=CNH–) that is contained in IL-1β, the relative intensity increase for the –NH bond can be presumed to be due to an indirect influence of the amino groups in IL-1β. Based on the above observation, the intensity level changes in the N 1s component in the XPS spectra on the 4-AMP/PEDOT and the IL-1β-templated PEBT/4-AMP/PEDOT films indicate an apparent surface-imprinting state on the polymer matrix surface via consecutive electropolymerization processes.

### 3.6. Electrochemical Performance for MIP Biosensor

The electrochemical response of the stepwise-modified surfaces of the SPCEs at each fabrication step was investigated by CV and EIS using a [Fe(CN)_6_]^3−/4−^ redox probe (Figure 3d,f). As plotted in Figure 3d, two typical redox peaks of the step-by-step-modified SPCEs were observed in a PBS solution (pH = 7.4) containing 5 mM of [Fe(CN)_6_]^3−/4−^. As the pretreatment process for the SPCE progressed using an acidic solvent (i.e., H_2_SO_4_, 0.5 M), the SPCE surface oxidized with the removal of impurities (i.e., adsorbed substances and organic binders), and therefore the peak current slightly decreased compared with that of the bare SPCE. This improvement may also be due to the hydrophilicity of the surface and carboxyl functional groups on the surface with the removal of surface contaminants, improving the electrochemical reversibility through mediated electron transfer reactions and increased carbon site density [60]. After the electrochemical deposition of the PEDOT film (marked in a red line), the anodic and cathodic peak currents increased due to the high electrical conductivity. Here, the PEDOT-deposited electrode showed a quasi-reversible electrochemical response with a Δ*E*_p_ of 0.15 V and Δ*I*_p_ of 0.367 mA, respectively. Right after the application of the 4-AMP layer, PEBT was finally electropolymerized and tested (marked in a green line), where decreases in the charge transfer were observed as a Δ*E*_p_ of 0.84 V and Δ*I*_p_ of 0.102 mA, respectively. The change in the peak currents indicates that the electron transfer rate could be adjusted between the electrode’s surface and the [Fe(CN)_6_]^3−/4−^ solution from the reduced conductivity by adding the PEBT matrix. To determine the imprinting efficiency, an unimprinted NIP was also prepared following the same procedure as a control sample in the absence of template molecules. By forming a selective recognition site after the removal of the IL-1β template (marked in a blue line), the empty imprinted cavity acts as an ion channel for redox probes to reach the electrolyte/MIP interface with the changed values as a Δ*E*_p_ of 0.34 V and Δ*I*_p_ of 19.573 mA. Eventually, the anodic peak was restored toward 0.33 V. In contrast, the NIP-based electrode presented a similar electrochemical response after exposure to a strong base environment utilized for template removal (Figure S14). This is because the PEBT films electropolymerized without templates did not contain imprinted sites, which restricted the electron transport of the redox probe. Therefore, this observation implies that the interaction strength of the MIP films by selective recognition cavities is much better than that of NIP films, although they have the same composition. 

The electrochemical behavior of the MIP-based electrode for each fabrication step was further characterized via EIS, as displayed in Figure 3e. Here, for all the EIS measurements, impedance spectra were recorded with a 10 mV amplitude and 0.01–100 kHz frequency range at 0.1 V potential, and Nyquist plot semicircles were fitted using the Randles equivalent circuit model. As marked in the red line in Figure 3e, PEDOT film electropolymerized on the activated SPCE significantly decreased the electron transfer resistance. However, the *R*_ct_ value increased after the imprinting process because the layer of PEBT with the IL-1β template disrupted the electron transport to the electrode surface (marked in a green line with a *R*_ct_ of 38.9 kΩ). Therefore, to quantify binding sites on MIPs, surface-imprinting techniques offer an effective means to create a recognition area. In our approach, we optimized the *R*_ct_ value of the PEBT through adjustments in the number of CV cycles and the monomer/template ratio during electropolymerization. The discernible differences in the impedance characteristics between the imprinted and extracted MIP films guided our selection of an optimal MIP film thickness, simultaneously ensuring the effective removal of the imprinted target material. The effective extraction of protein templates via surface imprinting can lead to sensitive responses in the performances of rebinding-based sensing applications [81,82,83]. Thus, the removal of IL-1β templates from the MIP matrix facilitated the diffusion of the redox probe through the polymeric film, decreasing the *R*_ct_ value to 8.8 kΩ (marked as a blue line). By the following rebinding target protein (i.e., 500 pg mL^−1^ IL-1β) captured in imprinted cavities in the MIP surface, the *R*_ct_ value was increased by 16.2 kΩ due to a block of the electron transfer by probe ions (marked as an orange line). In addition, the different electrochemical responses between the MIP and NIP films to the target molecules were evaluated under the same sequential procedures. As displayed in Figure 3f, the electron transfer resistance of the NIP film was rarely influenced by the presence of the target protein. As is well known in MIP approaches, MIP binding sites can be classified into distinct categories: specific/nonspecific adsorption and physical adsorption. In principle, specific adsorption exclusively binds the target substance, whereas nonspecific binding has the propensity to non-selectively bind molecules capable of forming hydrogen bonds with the MIP surface [84]. Separately, the target substance can be subject to physical adsorption on the surface. Based on these electrochemical behaviors of MIP and NIP films, as presented in Figure 3e,f, it was obviously demonstrated that our MIP-based biosensing systems were successfully constructed in integration with specific recognition sites that adsorb the target IL-1β protein, exhibiting a negligible adsorption trend compared to the MIPs. 

### 3.7. Analytical Characterization: POCT Demonstration

**Figure 4:**
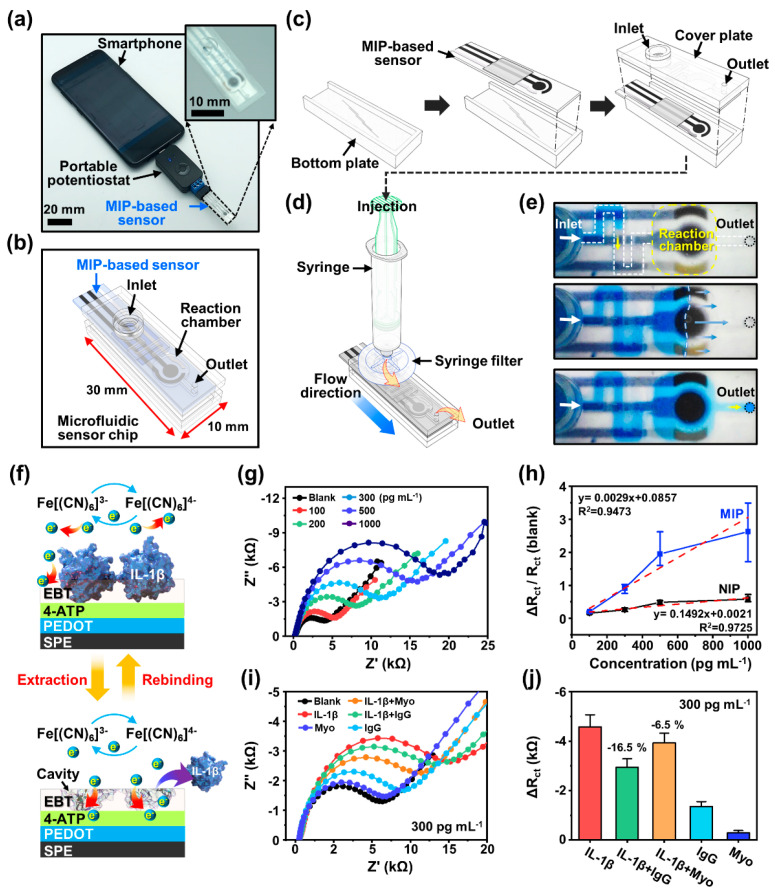
Smartphone-based POCT device integrated with MIP-based sensor for electrochemical measurement of IL-1β protein. (**a**) Photograph of a portable potentiostat combined with a microfluidic chip, connected to a smartphone via a USB-C connection to record signals via an application program. (**b**) Configuration of the fluidic channel chip plugged with MIP sensor. (**c**) A schematic of the assembly process of the fluidic cartridge and MIP electrode. (**d**) An illustration of the sample-loading step into the fluidic chip. (**e**) Photograph of the flowing test solution in the fluidic channel, reaching the reaction chamber. (**f**) Working mechanism of the electrochemical MIP-based sensor in the electrolyte. (**g**) EIS characteristics of the MIP-based sensor with different concentrations from 100 to 1000 pg mL^−1^. (**h**) Normalized Δ*R*_ct_/*R*_ct(blank)_ values as a function of the IL-1β concentration. (**i**) Evaluation of the interference responses of the MIP-based sensor to IL-1β protein mixed with other proteins (i.e., Myo and IgG) at the concentration of 300 pg mL^−1^. (**j**) Δ*R*_ct_ data obtained by converting the EIS results from (**i**).

Figure 4 illustrates a mobile POCT platform designed with an MIP-based biosensor to detect IL-1β. In this experimental scheme, we used a smartphone installed with a customized application program for data collection and transfer, highlighting the simplicity of the sensing approach for an engineered portable diagnostic device. Figure 4a shows the smartphone connected to a small-scale potentiostat module, as a reading interface, for the precise quantitative measurement of the biofluid via an MIP-based biosensor. To fully utilize the MIP sensor, we adopted a well-established microfluidic chip, simply produced via a stereolithographic 3D printer, to strictly guide the biofluid sample right onto the sensing electrode, as shown in the inset digital image of Figure 4a. In detail, the microfluidic chip was designed to induce a restricted flow from the inlet to the outlet in the channel, including a reaction chamber for sample processing with dimensions of 30 mm × 10 mm × 5 mm (length × width × height), which can be mounted on the MIP sensor strip (Figure 4b). Figure 4c schematically illustrates the assembly process for a microfluidic sensor chip, which consists of a bottom plate that holds the MIP sensor strip and a top fluidic component. Precise joints between each component ensure a secure bond without the need for adhesive, preventing solution leakage. This simple design offers the advantage of maintaining a constant fluidic flow via the engaged pressure from the inlet, primarily correlated with the contact angle of the injected liquid on the channel material; the size of the inlet and outlet was 13 mm in diameter to fit the syringe end, and the channel was configured to be 1 mm wide with a 2 mm height. In Figure 4d, the conceptual illustration demonstrates that injecting the liquid solution into the channel allows for a continuous flow of filtered biofluid (PTFE syringe filter, 0.45 μm) toward the reaction chamber located on the electrodes by the specific pressure from the syringe. Importantly, as displayed in Figure 4e, the transparent microfluidic channel-equipped MIP sensor chip was featured in an easy observation of the internal liquid via a pressure-driven reaction. For this demonstration, a dye-containing water solution was injected into the fluidic channel to visualize the stream of the propagating solution front so that we could estimate the flow rate with a constant syringe pressure. This experiment confirmed that the fluid flow was stable enough to fill the entire reaction chamber positioned on the MIP biosensor without forming bubbles. Thus, we expected a uniform contact between the liquid sample and the MIP electrode’s surface, which enables reliable impedance measurement with a minimal injection of analytes (~100 μL).

Based on the established PEBT-based MIP matrix, Figure 4f illustrates a selective recognition system in the surface-MIP structure to recognize the specific IL-1β cytokine by providing rebinding sites in the suggested operational mechanism as an electrochemical sensor, ultimately for quantitative detection. As described earlier, by immobilization with a target protein (upper panel) and the subsequent extraction of the template (lower panel) in the MIP matrix, high recognition ability and selectivity can be provided with copious rebinding sites (i.e., cavities) on the sensing electrode, at which the electron transfer in an aqueous environment (i.e., buffer solution/electrolyte/analytes) readily derives amperometric signal changes by the chemically complemental interactions. Figure 4g demonstrates the practical recombination ability of the molecular biosensor, which yielded concentration-dependent impedance responses when the standard samples of the IL-1β dispersed in PBS buffer solution and electrolytes (i.e., 5 mM [Fe(CN)_6_]^3−/4−^) were exposed on the MIP electrode surface. A selective binding affinity was investigated via EIS analysis at various concentration ranges (100–1000 pg mL^−1^) through the fluidic channel kit, where the IL-1β-containing solution was slowly infiltrated into the reaction chamber located at the MIP-integrated electrode. At the MIP electrode interface, the analyte solution was maintained for 1 h to measure the impedance changes. As appeared in the Nyquist plot, the semicircle diameters (i.e., *R*_ct_ values of the MIP film) were progressively increased with the increased concentration of the IL-1β samples, reflecting the higher resistance values by blocking the electron transfer from the electrolyte to the MIP electrode surface due to the spontaneous rebinding at the cavities. In other words, the *R*_ct_ values recorded after the recombination process represent the resistance of the electron transfer on the WE according to the ranged IL-1β concentration. With this hindered redox probe diffusion from the immobilized target protein, the rebinding of the adsorbed IL-1β was approximately proportional to the increased resistance, representing an effective recognition of individual proteins in the imprinted cavities. In contrast, the impedance signal responses before/after the incubation of the IL-1β samples at the NIP electrodes were found to be negligible, with minimal *R*_ct_ value changes, attributed to the nonspecific and unrecognizable physical adsorption of the IL-1β molecules. For more information, the relative changes in the *R*_ct_ values for the MIP or NIP electrodes were recorded in the presence of redox probe ions after each incubation. Based on the collected data, the results were calculated using the equation (i.e., Δ*R*_ct_/*R*_ct(Blank)_) shown in Figure 4h. Here, the *R*_ct(Blank)_ signifies the initial resistance when the IL-1β protein was extracted from the MIP matrix. Within this criteria, the impedimetric Δ*R*_ct_/*R*_ct(Blank)_ values for the MIP electrodes exhibited a significant increase in slope at the different IL-1β concentrations in the range of 100–1000 pg mL^−1^. The calibration curve for the MIP-equipped sensors showed a linear relationship with a correlation coefficient of [R^2^] = 0.9725. However, for the NIP electrodes, a gradual change in the slope was measured at the same concentration ranges, which obviously displayed a lower correlation coefficient ([R^2^] = 0.9473), although a linear relationship was observed. This direct comparison of the sensing capabilities implies that the successful IL-1β-imprinted cavities were sufficiently configured as rebinding sites that were mostly on the surface of the MIP matrix. Subsequently, from the calculated curves, the limit of detection (LOD) and the limit of quantitation (LOQ) for the MIP-based biosensor were estimated using *k* × S m^−1^; here, the S and m are the standard deviations of the y-intercept and the sensitivity, respectively. Each calculated LOD and LOQ was 252 and 765 pg mL^−1^ from the slope of the calibration curve, with *k* equal to the signal-to-noise ratio for the LOD (*k* = 3) and LOQ (*k* = 10). The summarized EIS measurements in our materials system indicate that the sensitivity of the electropolymerized MIP film on the SPCEs is in a wide range capable of selectively detecting IL-1β protein, confirming that only the IL-1β-imprinted electrodes can cause a clear shift in the impedimetric responses. Although some nonspecific binding of IL-1β was inevitable via random physical adsorption on the surface of the NIP electrode, these interfaces produced minimal interference in the electron transfer. 

In addition, to assess the specific binding of the IL-1β-imprinted MIP film, the selectivity was also evaluated via EIS analysis after immersing the electrodes in the PBS buffer solution with redox probe ions, containing other protein species, either individual or in a mixture. As for the interfering proteins, we selected myoglobin (Myo) (MW: 14 kDa; pl: 7.36), smaller than IL-1β, and immunoglobulin G (IgG) (MW: 150 kDa; pl: 7.5–7.8), larger but with a similar isoelectric point (pI) [38]. When it comes to the performance of a biosensor, it is important to consider the pI associated with the charge of the protein and how changes in the solution pH may affect this charge. As previously reported [85,86,87], when the pH of the solution deviates from its pI, proteins can carry a net positive charge at a lower pH or a net negative charge at a higher pH; this pH-dependent charge state significantly affects the binding and interaction of proteins with the MIP electrode surface. For example, the charge state of interfering proteins, such as Myo and IgG, affect the binding affinity on the sensing electrodes by changing the molecular interaction in the specific environments designed to detect specific biomarkers of IL-1β. Figure 4i displays the EIS responses obtained by separately measuring the solution samples of IL-1β, Myo, IgG, IL-1β/Myo, and IL-1β/IgG. Varied *R*_ct_ levels were detected in the same concentration range (i.e., 300 pg mL^−1^). Despite the small variations in the experimental results, the *R*_ct_ values from the single proteins of Myo and IgG were negligible, compared to the control group of the IL-1β samples, demonstrating their specific ability for excellent selective detection characteristics. However, as shown in Figure 4j, the presence of the Myo and IgG in the test solution slightly degraded the detection performance of the MIP sensor by ~16.5% and ~6.5% for the IgG and Myo, respectively, which was due to the interference in the rebinding of the IL-1β on the MIP electrodes. These results demonstrate that MIP-based biosensors can effectively detect the standard IL-1β cytokine in a certain range of concentrations (Figure 4g,h), underscoring their capability as a diagnostic tool. However, the screening of mixed-analyte samples was not fully established. Although a perfect molecular capture at the MIP-enabled cavity was limited, the presented results with the appropriated architecture indicated an affordable level at this stage with the MIP approach that enabled the selective detection of IL-1β biomarkers in the aqueous measurement condition, even in the presence of interfering proteins. In the near future, the development of biosensors with the multi-templating of target molecules on MIP surfaces may solve this problem, and such studies are currently underway in this community [88,89,90].

## 4. Conclusions

In summary, we have successfully engineered an MIP-based biosensor through electrochemical polymerization on customizable SPCEs for the precise detection of the cytokine IL-1β. The SPCEs were designed in a flexible format, featuring a simple three-channel electrode, which is a widely adopted configuration for electrochemical sensors. To serve as a highly effective molecular sensing device, we meticulously engineered the WE region with the MIP films. This entailed a two-step polymerization process, resulting in the endowment of robust chemical selectivity, minimal impedance drift, and stability within specific concentration ranges. As part of the layering process, we initiated the electropolymerization of EDOT and subsequently established a 4-AMP layer. Following this, the MIP films were polymerized simultaneously with EBT monomers and IL-1β templates by careful controlling the monomer/template ratio. The subsequent removal of the IL-1β templates from the MIP matrix allowed for the creation of chemically isolated cavities (i.e., rebinding sites) that act as artificial receptors, akin to “plastic antibodies”. This strategic architecting of the structured MIP electrodes enabled a stable detection of the IL-1β biomarker during the contact with the test solution of analytes. Our optimized synthesis method enabled a comprehensive investigation of the electrochemical behaviors via CV and EIS analyses. The collective data set confirmed the highly responsive nature of the PEBT-based MIP sensors, which were also subjected to separate comparisons with control samples of NIP films. To promote the practicality of immediate diagnosis, we present a mobile POCT device featuring an intuitive and user-friendly interface, which facilitates compatibility between MIP-based electrodes and smartphones. The disposable biosensors, linked to the mobile POCT device, demonstrated a reasonable sensitivity of a ~252 pg mL^−1^ LOD and ~765 pg mL^−1^ LOQ over IL-1β concentration ranges spanning from 100 to 1000 pg mL^−1^, with the help of the fluidic channel cartridge. The presented MIP-based biosensors exhibited high selectivity for the IL-1β biomarker, with minimal interference from structurally similar compounds in the test solution, such as Myo and IgG. Conclusively, our developed mobile platform, integrated with MIP-enabled electrodes, delivered a reliable electrochemical performance with a commendable detection capability, demonstrating the quantification of the cytokine levels. This collective set of results not only represents a value-added pathway toward the potential adoption of IVD methods for periodontitis detection, but it also suggests the potential for extending the developed diagnostic research to the real-time monitoring of other biomolecules.

## Data Availability

Data are contained within the article or Supplementary Materials.

## Supplementary Materials

The following are available online at https://www.mdpi.com/article/10.3390/bios13121013/s1, Figure S1: (a) Digital image of a screen-printed mask for printing a three-channel electrode pattern; the opening areas are only exposed to the carbon ink. (b) Optical image showing the channel region defined in the screen mask; Figure S2: SEM images representing the surface morphologies of screen-printed working and counter carbon electrodes with Ag/AgCl reference; Figure S3. The Nyquist plot (a) and corresponding *R*_ct_ value (b) after pretreatment of customized SPCEs; the pretreatment was performed using CV under a −0.65-1.05 V potential range, 50 mV s^−1^ scan rate, and 10 cycles in 0.5 M H_2_SO_4_ solution; Figure S4: The Nyquist plot (a) and CV voltammogram (b) after electrochemical deposition of PEDOT on SPCE surface; each PEDOT film was electropolymerized using CA under a fixed potential (10 s at 0.9 V) in 0.1 M PBS buffer; Figure S5: Schematic diagram of an MIP-based biosensor fabricated on an SPCE, sequentially composed of a PEDOT film, 4-AMP monolayer, and PEBT film; Figure S6: Consecutive CV curves obtained during the electrodeposition of EBT on the 4-AMP/PEDOT/SPCE (a) with and (b) without IL-1β templates; Figure S7: Electrochemical response of PEDOT film on SPCE surface electropolymerized by adjusting deposition voltage (a) and time (b); each peak current was obtained using CV under the parametric condition of a -0.3–0.1 V potential range and 100 mV s^−1^ scan rate in PBS solution (pH = 7.4) containing 5 mM of [Fe(CN)_6_]^3−/4−^; Figure S8: (a) Schematic illustration representing the correlation between the thickness of the MIP matrix and the reliability of the MIP-based biosensor: (i) incomplete imprinting, (ii) entrapped IL-1β templates by a thick PEBT film, and (iii) the formation of the optimized MIP thickness adjustment for IL-1β. The influence of the number of polymerization cycles (b) and the monomer/template concentration ratios (c) to survey the impedimetric response of the PEBT-based MIP sensor to IL-1β; Figure S9: The docking structures (i.e., before hydrogen bond formation) of the amino acid (alanine) templates with EBT monomer, describing the sites of dipole (δ^−^)–dipole (δ^+^) interactions. The heatmap displays the electrostatic potential mapping on the isovalue surface at a total electron density of 0.0004 using the self-consistent field method. Because the continuous carbon skeleton of the amino acid is connected to the terminal group, a neutral molecular orbital is not formed for a single sequence. Therefore, in the simulation, the terminals of the amino acid chain were hydrogenated to assume a neutral structure; Figure S10: The normalized Δ*R*_ct_/*R*_ct(blank)_ values as a function of the incubation time were derived from Nyquist plots obtained from the dynamic EIS responses for the MIP films. *R*_ct(blank)_ signifies the measured *R*_ct_ value of the MIP film before incubation in the specific concentration of IL-1β standard solution; Figure S11: SEM images showing the surface morphology of NIP films fabricated on 4-AMP/PEDOT/SPCE; Figure S12: AFM images for topological features of the MIP film (a) before and (b) after the extraction of IL-1β templates.; Figure S13: High-resolution XPS spectra of (a) PEDOT/SPCE and (b) 4-AMP/PEDOT/SPCE before the electrodeposition of EBT with IL-1β template; Figure S14: Electrochemical behaviors of NIP/4-AMP/PEDOT/SPCE according to the stepwise-fabrication process measured using CV in 0.01 M PBS (pH 7.2) containing the 5 mM [Fe (CN)_6_]^3−/4−^ redox probe; Table S1: Series of the ink types, according to the ratio of graphite flakes/carbon particles and the specific viscosity; Table S2: Hydrogen bond parameters between the EBT and the 16 amino acid molecules. Phenylalanine (Phe), Arginine (Arg), Leucine (Leu), and Valine (Val) are only located in the secondary structures, such as the β-sheet structure; Table S3: Binding energy between EBT and the amino acid in protein sequence of IL-1β; Table S4: Atomic composition and concentration of stepwise-modified surfaces of SPCEs at each fabrication step.

## References

[1] Kinane D.F., Stathopoulou P.G., Papapanou P.N. (2017). Periodontal Diseases. Nat. Rev. Dis. Primers.

[2] Kassebaum N.J., Bernabé E., Marcenes W. (2014). Global Burden of Severe Periodontitis in 1990–2010: A Systematic Review and Meta-Regression. J. Dent. Res..

[3] Loos B.G., Craandiji J., Hoek F.J., Wertheim-van Dillen P.M.E., van der Velden U. (2000). C-Reactive Protein and Other Markers of Systemic Inflammation in Relation to Cardiovascular Diseases are Elevated in Periodontitis. J. Periodontol..

[4] Socransky S.S., Haffajee A.D., Cugini M.A., Smith C., Kent R.L. (1998). Microbial Complexes in Subgingival Plaque. J. Clin. Periodontol..

[5] Holt S.C., Ebersole J.L. (2005). Porphyromonas Gingivalis, Treponema Denticola, and Tannerella Forsythia: The ‘Red Complex’, a Prototype Polybacterial Pathogenic Consortium in Periodontitis. Periodontology 2000.

[6] Hajishengallis G. (2015). Periodontitis: From Microbial Immune Subversion to Systemic Inflammation. Nat. Rev. Immunol..

[7] Dominy S.S., Lynch C., Ermini F., Benedyk M., Marczyk A., Konradi A., Nguyen M., Haditsch U., Raha D., Griffin C. (2019). Porphyromonas Gingivalis in Alzheimer’s Disease Brains: Evidence for Disease Causation and Treatment with Small-Molecule Inhibitors. Sci. Adv..

[8] Armitage G.C. (2004). Periodontal Diagnoses and Classification of Periodontal Diseases. Periodontology 2000.

[9] Lequin R.M. (2005). Enzyme Immunoassay (EIA)/Enzyme-Linked Immunosorbent Assay (ELISA). Clin. Chem..

[10] Li Y.-F., Sun Y.-M., Beier R.C., Lei H.-T., Gee S., Hammock B.D., Wang H., Wang Z., Sun X., Shen Y.-D. (2017). Immunochemical Techniques for Multianalyte Analysis of Chemical Residues in Food and the Environment: A Review. Trac. Trends Anal. Chem..

[11] Juncker D., Bergeron S., Laforte V., Li H. (2014). Cross-Reactivity in Antibody Microarrays and Multiplexed Sandwich Assays: Shedding Light on the Dark Side of Multiplexing. Curr. Opin. Chem. Biol..

[12] Park R., Jeon S., Jeong J., Park S.-Y., Han D.-W., Hong S.W. (2022). Recent Advances of Point-of-Care Devices Integrated with Molecularly Imprinted Polymers-Based Biosensors: From Biomolecule Sensing Design to Intraoral Fluid Testing. Biosensors.

[13] Luppa P.B., Müller C., Schlichtiger A., Schlebusch H. (2011). Point-of-Care Testing (POCT): Current Techniques and Future Perspectives. TrAC Trends Anal. Chem..

[14] Zhang J.J., Lan T., Lu Y. (2020). Translating In Vitro Diagnostics from Centralized Laboratories to Point-of-Care Locations using Commercially-Available Handheld Meters. TrAC Trends Anal. Chem..

[15] Ahn C.H., Choi J.-W., Beaucage G., Nevin J.H., Lee J.B., Puntambekar A., Lee J.Y. (2004). Disposable Smart Lab on a Chip for Point-of-Care Clinical Diagnostics. Proc. IEEE.

[16] Jeon S., Kwon Y.W., Park J.Y., Hong S.W. (2019). Fluorescent Detection of Bovine Serum Albumin using Surface Imprinted Films Formed on PDMS Microfluidic Channels. J. Nanosci. Nanotechnol..

[17] Liu Y., Zhan L., Qin Z., Sackrison J., Bischof J.C. (2021). Ultrasensitive and Highly Specific Lateral Flow Assays for Point-of-Care Diagnosis. ACS Nano.

[18] Dighe K., Moitra P., Alafeef M., Gunaseelan N., Pan D. (2022). A Rapid RNA Extraction-Free Lateral Flow Assay for Molecular Point-of-Care Detection of SARS-CoV-2 Augmented by Chemical Probes. Biosens. Bioelectron..

[19] Contreras-Naranjo J.E., Aguilar O. (2019). Suppressing Non-Specific Binding of Proteins onto Electrode Surfaces in the Development of Electrochemical Immunosensors. Biosensors.

[20] Volpe G., Moscone D., Ricci F., Piermarini S., Palleschi G., Arduini F., Micheli L. (2016). Electrochemical Biosensors Based on Nanomodified Screen-Printed Electrodes: Recent Applications in Clinical Analysis. TrAC Trends Anal. Chem..

[21] Xu K., Zhou R., Takei K., Hong M. (2019). Toward Flexible Surface-Enhanced Raman Scattering (SERS) Sensors for Point-of-Care Diagnostics. Adv. Sci..

[22] Hang Y., Boryczka J., Wu N. (2021). Visible-Light and Near-Infrared Fluorescence and Surface-Enhanced Raman Scattering Point-of-Care Sensing and Bio-Imaging: A Review. Chem. Soc. Rev..

[23] Kim S.H., Jeon S., Yoo D., Zhang M., Park W., Kang K., Choi C., Hong S.W., Lee D. (2023). Guided Wrinkling of Hierarchically Structured Nanoporous Gold Films for Improved Surface-Enhanced Raman Scattering Performance. Adv. Mater. Interfaces.

[24] Shrivastava S., Trung T.Q., Lee N.-E. (2020). Recent Progress, Challenges, and Prospects of Fully Integrated Mobile and Wearable Point-of-Care Testing Systems for Self-Testing. Chem. Soc. Rev..

[25] Jeun M., Lee H.J., Park S., Do E.J., Choi J., Sung Y.N., Hong S.M., Kim S.Y., Kim D.H., Kang J.Y. (2019). A Novel Blood-Based Colorectal Cancer Diagnostic Technology Using Electrical Detection of Colon Cancer Secreted Protein-2. Adv. Sci..

[26] Chen L., Wang X., Lu W., Wu X., Li J. (2016). Molecular Imprinting: Perspectives and Applications. Chem. Soc. Rev..

[27] Potyrailo R.A., Mirsky V.M. (2008). Combinatorial and High-Throughput Development of Sensing Materials: The First 10 Years. Chem. Rev..

[28] Vlatakis G., Andersson L.I., Muller R.S., Mosbach K. (1993). Drug Assay using Antibody Mimics Made by Molecular Imprinting. Nature.

[29] Poma A., Guerreiro A., Whitcombe M.J., Piletska E.V., Turner A.P.F., Piletsky S.A. (2013). Solid-Phase Synthesis of Molecularly Imprinted Polymer Nanoparticles with a Reusable Template—“Plastic Antibodies”. Adv. Funct. Mater..

[30] Yang J.C., Hong S.W., Park J. (2021). Improving Surface Imprinting Effect by Reducing Nonspecific Adsorption on Non-Imprinted Polymer Films for 2,4-D Herbicide Sensors. Chemosensors.

[31] Haupt K., Medina Rangel P.X., Bui B.T.S. (2020). Molecularly Imprinted Polymers: Antibody Mimics for Bioimaging and Therapy. Chem. Rev..

[32] Jeon S., Park R., Jeong J., Heo G., Lee J., Shin M.C., Kwon Y.W., Yang J.C., Park W.I., Kim K.S. (2021). Rotating Cylinder-Assisted Nanoimprint Lithography for Enhanced Chemisorbable Filtration Complemented by Molecularly Imprinted Polymers. Small.

[33] Sellergren B., Allender C.J. (2005). Molecularly Imprinted Polymers: A Bridge to Advanced Drug Delivery. Adv. Drug Deliv. Rev..

[34] He W., You M., Wan W., Xu F., Li F., Li A. (2018). Point-of-Care Periodontitis Testing: Biomarkers, Current Technologies, and Perspectives. Trends Biotechnol..

[35] Ayankojo A.G., Reut J., Boroznjak R., Öpik A., Syritski V. (2018). Molecularly imprinted poly(meta-phenylenediamine) based QCM sensor for detecting Amoxicillin. Sens. Actuators B Chem..

[36] Wang M., Yang Y., Min J., Song Y., Tu J., Mukasa D., Ye C., Xu C., Heflin N., McCune J.S. (2022). A Wearable Electrochemical Biosensor for the Monitoring of Metabolites and Nutrients. Nat. Biomed. Eng..

[37] Ayankojo A.G., Boroznjak R., Reut J., Opik A., Syritski V. (2022). Molecularly Imprinted Polymer based Electrochemical Sensor for Quantitative Detection of SARS-CoV-2 Spike Protein. Sens. Actuators B Chem..

[38] Cardoso A.R., de Sá M.H., Sales M.G.F. (2019). An Impedimetric Molecularly-Imprinted Biosensor for Interleukin-1β Determination, Prepared by In-Situ Electropolymerization on Carbon Screen-printed Electrodes. Bioelectrochemistry.

[39] Cheng R., Wu Z., Li M., Shao M., Hu T. (2020). Interleukin-1β is a Potential Therapeutic Target for Periodontitis: A Narrative Review. Int. J. Oral Sci..

[40] Kobayashi M., Squires G.R., Mousa A., Tanzer M., Zukor D.J., Antoniou J., Feige U., Poole A.R. (2005). Role of Interleukin-1 and Tumor Necrosis Factor α in Matrix Degradation of Human Osteoarthritic Cartilage. Arthritis Rheumatol..

[41] Offenbacher S., Barros S.P., Singer R.E., Moss K., Williams R.C., Beck J.D. (2007). Periodontal Disease at the Biofilm–Gingival Interface. J. Periodontol..

[42] Dinarello C.A. (2011). Interleukin-1 in the Pathogenesis and Treatment of Inflammatory Diseases. Blood.

[43] Denes A., Pinteaux E., Rothwell N.J., Allan S.M. (2011). Interleukin-1 and Stroke: Biomarker, Harbinger of Damage, and Therapeutic Target. Cerebrovasc. Dis..

[44] Crapnell R.D., Hudson A., Foster C.W., Eersels K., Grinsven B.V., Cleij T.J., Banks C.E., Peeters M. (2019). Recent Advances in Electrosynthesized Molecularly Imprinted Polymer Sensing Platforms for Bioanalyte Detection. Sensors.

[45] Erdőssy J., Horváth V., Yarman A., Scheller F.W., Gyurcsányi R.E. (2016). Electrosynthesized Molecularly Imprinted Polymers for Protein Recognition. TrAC Trends Anal. Chem..

[46] Tavares A.P., Sales M.G.F. (2018). Novel Electro-Polymerized Protein-Imprinted Materials using Eriochrome Black T: Application to BSA Sensing. Electrochim. Acta.

[47] Marques A.C., Cardoso A.R., Martins R., Sales M.G.F., Fortunato E. (2020). Laser-Induced Graphene-Based Platforms for Dual Biorecognition of Molecules. ACS Appl. Nano Mater..

[48] Culver H.R., Peppas N.A. (2017). Protein-Imprinted Polymers: The Shape of Things to Come?. Chem. Mater..

[49] Whitcombe M.J., Chianella I., Larcombe L., Piletsky S.A., Noble J., Porter R., Horgan A. (2011). The Rational Development of Molecularly Imprinted Polymer-Based Sensors for Protein Detection. Chem. Soc. Rev..

[50] Tu J., Torrente-Rodríguez R.M., Wang M., Gao W. (2019). The Era of Digital Health: A Review of Portable and Wearable Affinity Biosensors. Adv. Funct. Mater..

[51] Shanmugam N.R., Muthukumar S., Prasad S. (2016). Ultrasensitive and Low-Volume Point-of-Care Diagnostics on Flexible Strips-a Study with Cardiac Troponin Biomarkers. Sci. Rep..

[52] Wang J., Pedrero M., Sakslund H., Hammerich O., Pingarron J. (1996). Electrochemical Activation of Screen-Printed Carbon Strips. Analyst.

[53] Obaje E.A., Cummins G., Schulze H., Mahmood S., Desmulliez M.P.Y., Bachmann T.T. (2016). Carbon Screen-Printed Electrodes on Ceramic Substrates for Label-Free Molecular Detection of Antibiotic Resistance. J. Interdiscip. Nanomed..

[54] Liu L., Shen Z., Zhang X., Ma H. (2021). Highly Conductive Graphene/Carbon Black Screen Printing Inks for Flexible Electronics. J. Colloid Interface Sci..

[55] Pantea D., Darmstadt H., Kaliaguine S., Roy C. (2003). Electrical Conductivity of Conductive Carbon Blacks: Influence of Surface Chemistry and Topology. Appl. Surf. Sci..

[56] Potts S.J., Korochkina T., Holder A., Jewell E., Phillips C., Claypole T. (2022). The Influence of Carbon Morphologies and Concentrations on the Rheology and Electrical Performance of Screen-Printed Carbon Pastes. J. Mater. Sci..

[57] Phillips C., Al-Ahmadi A., Potts S.J., Claypole T., Deganello D. (2017). The Effect of Graphite and Carbon Black Ratios on Conductive Ink Performance. J. Mater. Sci..

[58] Fonseca W.T., Castro K.R., Oliveira T.R., Faria R.C. (2021). Disposable and Flexible Electrochemical Paper-Based Analytical Devices using Low-cost Conductive Ink. Electroanalysis.

[59] Cui G., Yoo J.H., Lee J.S., Yoo J., Uhm J.H., Cha G.S., Nam H. (2001). Effect of Pre-Treatment on the Surface and Electrochemical Properties of Screen-Printed Carbon Paste Electrodes. Analyst.

[60] Et Taouil A., Lallemand F., Hihn J.Y., Melot J.M., Blondeau-Patissier V., Lakard B. (2011). Doping Properties of PEDOT Films Electrosynthesized under High Frequency Ultrasound Irradiation. Ultrason. Sonochem..

[61] Sanchez-Sanchez A., del Agua I., Malliaras G.G., Mecerreyes D., Aguilar M.R., San Román J. (2019). Conductive Poly(3,4-Ethylenedioxythiophene) (PEDOT)-Based Polymers and Their Applications in Bioelectronics. Smart Polymers and Their Applications.

[62] Kim D., Franco-Gonzalez J.F., Zozoulenko I. (2021). How Long are Polymer Chains in Poly(3,4-ethylenedioxythiophene): Tosylate Films? An Insight from Molecular Dynamics Simulations. J. Phys. Chem. B.

[63] Liu J., Wei B., Sloppy J.D., Ouyang L., Ni C., Martin D.C. (2015). Direct Imaging of the Electrochemical Deposition of Poly(3,4-ethylenedioxythiophene) by Transmission Electron Microscopy. ACS Macro Lett..

[64] Pereira M.V., Marques A.C., Oliveira D., Martins R., Moreira F.T.C., Sales M.G.F., Fortunato E. (2020). Paper-Based Platform with an in Situ Molecularly Imprinted Polymer for β-Amyloid. ACS Omega.

[65] Yao H., Sun Y., Lin X., Tang Y., Huang L. (2007). Electrochemical characterization of poly(eriochrome black T) modified glassy carbon electrode and its application to simultaneous determination of dopamine, ascorbic acid and uric acid. Electrochim. Acta.

[66] Lucas X., Bauzá A., Frontera A., Quiñonero D. (2016). A thorough Anion–π Interaction Study in Biomolecules: On the Importance of Cooperativity Effects. Chem. Sci..

[67] Cysewska K., Karczewski J., Jasiński P. (2015). Influence of Electropolymerization Conditions on the Morphological and Electrical Properties of PEDOT Film. Electrochim. Acta.

[68] Lisdat F., Schäfer D. (2008). The Use of Electrochemical Impedance Spectroscopy for Biosensing. Anal. Bioanal. Chem..

[69] Choi D.Y., Yang J., Hong S., Park J. (2022). Molecularly Imprinted Polymer-Based Electrochemical Impedimetric Sensors on Screen-Printed Carbon Electrodes for the Detection of Trace Cytokine IL-1beta. Biosens. Bioelectron..

[70] Malitesta C., Mazzotta E., Picca R.A., Poma A., Chianella I., Piletsky S.A. (2012). MIP Sensors—The Electrochemical Approach. Anal. Bioanal. Chem..

[71] Riskin M., Tel-Vered R., Bourenko T., Granot E., Willner I. (2008). Imprinting of Molecular Recognition Sites through Electropolymerization of Functionalized Au Nanoparticles: Development of an Electrochemical TNT Sensor Based on π-Donor-Acceptor Interactions. J. Am. Chem. Soc..

[72] Choi D.Y., Yang J.C., Park J. (2022). Optimization and Characterization of Electrochemical Protein Imprinting on Hemispherical Porous Gold Patterns for the Detection of Trypsin. Sens. Actuators B Chem..

[73] Mukasa D., Wang M., Min J., Yang Y., Solomon S.A., Han H., Ye C., Gao W. (2023). A Computationally Assisted Approach for Designing Wearable Biosensors toward Non-Invasive Personalized Molecular Analysis. Adv. Mater..

[74] Tomasi J., Mennucci B., Cammi R. (2005). Quantum Mechanical Continuum Solvation Models. Chem. Rev..

[75] Mahony J.O., Nolan K., Smyth M.R., Mizaikoff B. (2005). Molecularly imprinted polymers—Potential and challenges in analytical chemistry. Sens. Anal. Chim. Acta.

[76] Chen W.-Y., Chen C.-S., Lin F.-Y. (2001). Molecular recognition in imprinted polymers: Thermodynamic investigation of analyte binding using microcalorimetry. J. Chromatogr. A.

[77] Clore G.M., Wingfield P.T., Gronenborn A.M. (1991). High-Resolution Three-Dimensional Structure of Interleukin 1 beta. in Solution by Three-and Four-Dimensional Nuclear Magnetic Resonance Spectroscopy. Biochemistry.

[78] Abo-Elmagd I.F., Mahmoud A.M., Al-Ghobashy M.A., Nebsen M., El Sayed N.S., Nofal S., Soror S.H., Todd R., Elgebaly S.A. (2021). Impedimetric Sensors for Cyclocreatine Phosphate Determination in Plasma Based on Electropolymerized Poly(o-phenylenediamine) Molecularly Imprinted Polymers. ACS Omega.

[79] Kim S.H., Rho Y., Cho E., Myung J.S., Lee S.-J. (2021). Surface plasmonic resonance tunable nanocomposite thin films applicable to color filters, heat mirrors, semi-transparent electrodes, and electromagnetic-shields. Nanoscale.

[80] Puerto M.A., Costa T.M.H., Jornada J.A.H., Balzaretti N.M. (2018). Pyrolisys of a-aminoacids under high-pressure investigated by XPS, Raman and infrared spectroscopy. Mater. Chem. Phys..

[81] Kalecki J., Iskierko Z., Cieplak M., Sharma P.S. (2020). Oriented Immobilization of Protein Templates: A New Trend in Surface Imprinting. ACS Sensors.

[82] Ertürk G., Mattiasson B. (2017). Molecular Imprinting Techniques Used for the Preparation of Biosensors. Sensors.

[83] Yang J.C., Shin H.-K., Hong S.W., Park J.Y. (2015). Lithographically Patterned Molecularly Imprinted Polymer for Gravimetric Detection of Trace Atrazine. Sens. Actuators B Chem..

[84] Zhongbo Z., Hu J. (2008). Selective removal of estrogenic compounds by molecular imprinted polymer (MIP). Water Res..

[85] Guo S., Zhu X., Jańczewski D., Lee S.S.C., He T., Teo S.L.M., Vancso G.J. (2016). Measuring protein isoelectric points by AFM-based force spectroscopy using trace amounts of sample. Nat. Nanotechnol..

[86] Pergande M.R., Cologna S.M. (2017). Isoelectric Point Separations of Peptides and Proteins. Proteomes.

[87] Tlili A., Attia G., Khaoulani S., Mazouz Z., Zerrouki C., Yaakoubi N., Othmane A., Fourati N. (2021). Contribution to the Understanding of the Interaction between a Polydopamine Molecular Imprint and a Protein Model: Ionic Strength and pH Effect Investigation. Sensors.

[88] Liu Y., Liu Y., Liu Z., Hill J.P., Alowasheeir A., Xu Z., Xu X., Yamauchi Y. (2021). Ultra-Durable, Multi-Template Molecularly Imprinted Polymers for Ultrasensitive Monitoring and Multicomponent Quantification of Trace Sulfa Antibiotics. J. Mater. Chem. B.

[89] Díaz-Álvarez M., Martín-Esteban A. (2021). Molecularly Imprinted Polymer-Quantum Dot Materials in Optical Sensors: An Overview of Their Synthesis and Applications. Biosensors.

[90] Johari-Ahar M., Karami P., Ghanei M., Afkhami A., Bagheri H. (2018). Development of a Molecularly Imprinted Polymer Tailored on Disposable Screen-Printed Electrodes for Dual Detection of EGFR and VEGF using Nano-Liposomal Amplification Strategy. Biosens. Bioelectron..

