# Supplementary material for: Mobile Point-of-Care Device Using Molecularly Imprinted Polymer-Based Chemosensors Targeting Interleukin-1β Biomarker
Source: Biosensors (Basel). 2023 Dec 5;13(12):1013. doi: 10.3390/bios13121013 (PMC10741793; doi:10.3390/bios13121013)
Supplement: Supplementary file 1 [file biosensors-13-01013-s001.zip › biosensors-2706744-supplementary.pdf]

Supporting Information

Mobile Point-of-Care Device Using Molecularly Imprinted Polymer-Based Chemosensors Targeting Interleukin-1β Biomarker

Rowoon Park [1,†], Sangheon Jeon [1,†], Jae Won Lee [1,†], Jeonghwa Jeong [1], Young Woo Kwon [2], Sung Hyun Kim [2], Joonkyung Jang [3] and Dong-Wook Han [1] and Suck Won Hong [1,2,*]

1 Department of Cogno-Mechatronics Engineering, Department of Optics and Mechatronics Engineering, College of Nanoscience and Nanotechnology, Pusan National University, Busan 46241, Republic of Korea; rowoon.p153@gmail.com (R.P.); jsfhse@pusan.ac.kr (S.J.); jik904@pusan.ac.kr (J.W.L.); jh.jeong@pusan.ac.kr (J.J.); nanohan@pusan.ac.kr (D.-W.H.)

2 Engineering Research Center for Color Modulation Extrasensory Cognitive Technology, Pusan National University, Busan 46241, Republic of Korea; handmade4522@gmail.com (Y.W.K.); ksh1332@pusan.ac.kr (S.H.K.)

3 Department of Nanoenergy Engineering, Pusan National University, Busan 46241, Republic of Korea; jkjang@pusan.ac.kr

* Correspondence: swhong@pusan.ac.kr

† These authors contributed equally to this work.

*Correspondence should be addressed to S.W.H. (email: swhong@pusan.ac.kr)

KEYWORDS: molecularly imprinted polymers, interleukin-1β, cytokine, electrochemical impedance spectroscopy, point-of-care testing

Table S1. Series of the ink types, according to the ratio of graphite flakes/carbon particles and the specific viscosity.

	Content of Graphite	Viscosity (Cps)
Type 1	~25%	40,000 ± 20000
Type 2	~21.4%	34,500 ± 17,000
Type 3	~18.8%	30,000 ± 15,000

Table S2. Hydrogen bond parameters between the EBT and the 16 amino acid molecules. Phenylalanine (Phe), Arginine (Arg), Leucine (Leu), and Valine (Val) are only located in the secondary structures, such as the β-sheet structure. It should be noted that these secondary structures are located inside the IL-1β protein, which does not participate in hydrogen bonds except for some terminal groups.

**Molecule structure**	**Energy of the spatial structure (Hartree)**	**Binding energy of amino acid-EBT ($kJ\ mol^{-1}$)**	**Hydrogen bond Length (Å)**	**Mulliken charge of targeting Oxygen (δ)**
**EBT**	**-1857.420101**	**free monomer**		
Alanine (Ala)	-248.422666	Template amino acid		
EBT-Ala (1)	-2105.837524	13.765	1.89	-0.420
EBT-Ala (2)	-2105.833687	23.840	1.95	-0.578
EBT-Ala (3)	-2105.813399	77.106	2.32	-0.655
EBT-Ala (4)	-2105.830806	31.404	1.55	-0.333
**EBT-Ala total**		**146.115**		
Aspartic Acid (Asp)	-436.346837	Template amino acid	-	-
EBT-Asp (1)	-2293.759472	19.602	2.03	-0.462
EBT-Asp (2)	-2293.736226	53.798	2.33	-0.527
EBT-Asp (3)	-2293.740248	80.635	2.62	-0.640
EBT-Asp (4)	-2293.746447	70.075	2.80	-0.285
**EBT-Asp total**		**224.110**		
Glutamic Acid (Glu)	-475.643492	Template amino acid	-	-
EBT-Glu (1)	-2333.058907	12.302	2.35	-0.526
EBT-Glu (2)	-2333.051405	32.000	2.57	-0.442
EBT-Glu (3)	-2333.039015	64.530	2.77	-0.590
EBT-Glu (4)	-2333.046408	45.121	2.31	-0.321
**EBT-Glu total**		**153.953**		
Glycine (Gly)	-209.100112	Template amino acid		
EBT-Gly (1)	-2066.510703	24.969	2.08	-0.408
EBT-Gly (2)	-2066.519264	2.492	2.13	-0.577
EBT-Gly (3)	-2066.512807	19.444	2.54	-0.630
EBT-Gly (4)	-2066.513079	18.730	2.21	-0.302
**EBT-Gly total**		**65.635**		
Asparagine (Asn)	-417.051539	Template amino acid		
EBT-Asn (1)	-2274.468174	9.100	2.49	-0.473
EBT-Asn (2)	-2274.457040	38.333	2.54	-0.451
EBT-Asn (3)	-2274.449125	59.113	2.67	-0.626
EBT-Asn (4)	-2274.441440	79.291	2.33	-0.280
**EBT-Asn total**		**185.837**		

Molecule structure	Energy of the spatial structure (Hartree)	Binding energy of amino acid-EBT (kJ mol^{-1})	Hydrogen bond Length (Å)	Mulliken charge of targeting Oxygen (δ)
Proline (Pro)	-325.795736	Template amino acid	-	-
EBT-Pro (1)	-2183.206075	25.630	2.25	-0.393
EBT-Pro (2)	-2183.215328	1.337	2.84	-0.583
EBT-Pro (3)	-2183.215603	0.615	2.22	-0.640
EBT-Pro (4)	-2183.212880	7.764	2.39	-0.311
**EBT-Pro total**		**35.345**		
Glutamine (Gln)	-456.345712	Template amino acid	-	-
EBT-Gln (1)	-2313.763244	**6.745**	2.38	-0.483
EBT-Gln (2)	-2313.734143	83.149	2.20	-0.597
EBT-Gln (3)	-2313.722483	113.764	2.14	-0.642
EBT-Gln (4)	-2313.754975	28.455	2.58	-0.289
**EBT-Gln total**		**232.113**		
Serine (Ser)	-323.584919	Template amino acid	-	-
EBT-Ser (1)	-2180.988028	44.612	2.42	-0.492
EBT-Ser (2)	-2180.982213	59.880	2.34	-0.617
EBT-Ser (3)	-2180.992089	33.950	2.44	-0.614
EBT-Ser (4)	-2181.003416	4.211	2.21	-0.305
**EBT-Ser total**		**142.653**		
Threonine (Thr)	-362.886784	Template amino acid	-	-
EBT-Thr (1)	-2220.300411	16.997	2.13	-0.669
EBT-Thr (2)	-2220.305235	4.332	2.76	-0.576
EBT-Thr (3)	-2220.294442	32.669	2.81	-0.642
EBT-Thr (4)	-2220.306882	0.008	2.66	-0.297
**EBT-Thr total**		**54.006**		
Lysine (Lys)	-422.096607	Template amino acid		
EBT-Lys (1)	-2279.493463	61.030	2.03	-0.625
EBT-Lys (2)	-2279.507590	23.940	2.15	-0.579
EBT-Lys (3)	-2279.491325	66.643	2.58	-0.629
EBT-Lys (4)	-2279.510807	15.493	2.15	-0.400
**EBT-Lys total**		**167.106**		
Tyrosine (Tyr)	-554.556305	Template amino acid		
EBT-Tyr (1)	-2411.964316	31.742	2.31	-0.649
EBT-Tyr (2)	-2411.957141	50.580	2.48	-0.586
EBT-Tyr (3)	-2411.965785	27.885	2.72	-0.639
EBT-Tyr (4)	-2411.962481	36.561	2.52	-0.284
**EBT-Tyr total**		**146.768**		

**Molecule structure**	**Energy of the spatial structure (Hartree)**	**Binding energy of amino acid-EBT (kJ mol^{-1})**	**Hydrogen bond Length (Å)**	**Mulliken charge of targeting Oxygen (*δ*)**
Methionine (Met)	-725.157334	Template amino acid	-	-
EBT-Met (1)	-2582.567942	24.924	2.63	-0.643
EBT-Met (2)	-2582.564610	33.672	2.65	-0.598
EBT-Met (3)	-2582.564858	33.021	2.65	-0.632
EBT-Met (4)	-2582.571693	15.076	2.43	-0.301
**EBT-Met total**		**106.693**		
Phenylalanine (Phe)	-479.383724	Template amino acid	-	-
EBT-Phe (1)	-2336.794185	25.310	4.32	-0.621
EBT-Phe (2)	-2336.803707	0.310	2.33	-0.440
EBT-Phe (3)	-2336.803673	0.401	5.62	-0.600
EBT-Phe (4)	-2336.803558	0.699	6.80	-0.301
**EBT-Phe total**		**26.72**		
Arginine (Arg)	-531.593043	Template amino acid	-	-
EBT-Arg (1)	-2389.006119	18.444	2.44	-0.664
EBT-Arg (2)	-2389.004026	23.940	2.47	-0.431
EBT-Arg (3)	-2388.980147	86.633	2.99	-0.607
EBT-Arg (4)	-2388.984379	75.523	3.24	-0.341
**EBT-Arg total**		**204.54**		
Leucine (Leu)	-366.306240	Template amino acid		
EBT-Leu (1)	-2223.718982	19.321	4.54	-0.637
EBT-Leu (2)	-2223.721631	12.365	4.31	-0.451
EBT-Leu (3)	-2223.725918	1.111	4.92	-0.632
EBT-Leu (4)	-2223.726051	0.761	5.40	-0.343
**EBT-Leu total**		**33.558**		
Valine (Val)	-323.216119	Template amino acid		
EBT-Val (1)	-2180.629608	17.361	3.11	-0.661
EBT-Val (2)	-2180.630982	13.752	2.87	-0.451
EBT-Val (3)	-2180.628172	21.131	3.07	-0.636
EBT-Val (4)	-2180.626796	24.744	3.42	-0.330
**EBT-Val total**		**76.988**		

Table S3. Binding energy between EBT and the amino acid in protein sequence of IL-1β.

**Amino acid**	**Abbreviation**	**Frequency**	**Active site**	**Binding energy (kJ mol^{-1})**
Alanine	Ala, A	5	2	146.115
Aspartic Acid	Asp, D	8	6	224.11
Glutamic Acid	Glu, E	11	6	153.953
Glycine	Gly, G	8	5	65.635
Asparagine	Asn, N	9	6	185.837
Proline	Pro, P	8	5	35.345
Glutamine	Gln, Q	12	10	232.113
Serine	Ser, S	14	2	142.653
Threonine	Thr, T	6	2	54.006
Lysine	Lys, K	15	6	167.106
Tyrosine	Tyr, Y	4	2	146.768
Methionine	Met, M	6	2	106.693
**Total frequency**		153	58	

Table S4. Atomic composition and concentration of stepwise-modified surfaces of SPCE at each fabrication step.

Sample	Atomic concentration (%)			
	C 1s	O 1s	N 1s	S 2p
PEDOT film	88.1	10.5	None	1.4
4-AMP/PEDOT film	82.0	10.6	4.7	2.7
PEBT/4-AMP/PEDOT film with IL-1β template	73.3	15.8	7.0	3.9

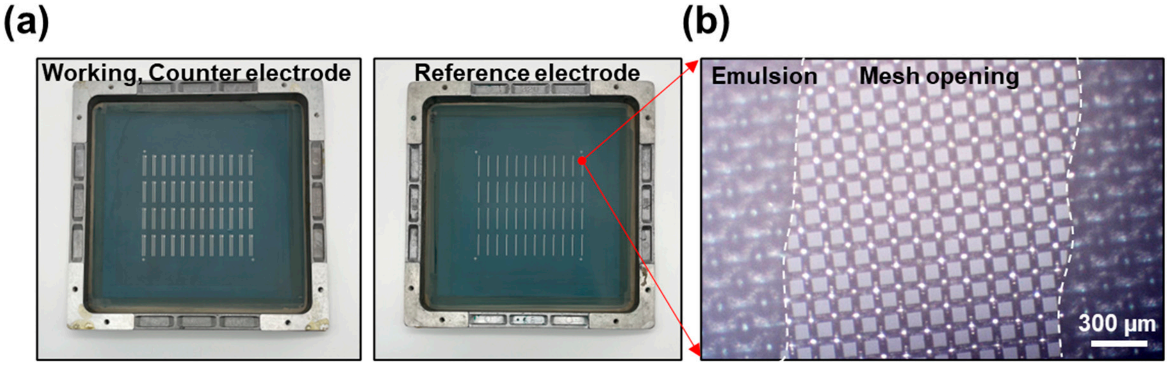

Figure S1. (a) Digital image of screen-printed masks for printing a three-channel electrode pattern; the opening area are only exposed to the carbon ink. (b) Optical image showing the channel region defined in the screen mask.

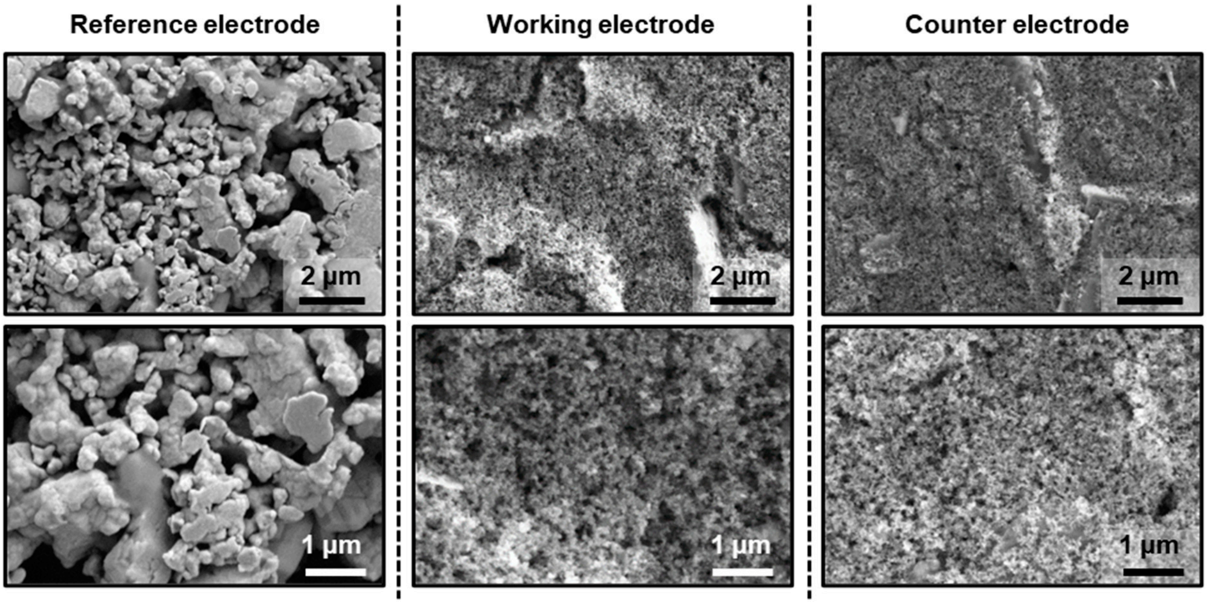

Figure S2. SEM images representing the surface morphology of screen-printed working and counter carbon electrodes with Ag/AgCl reference.

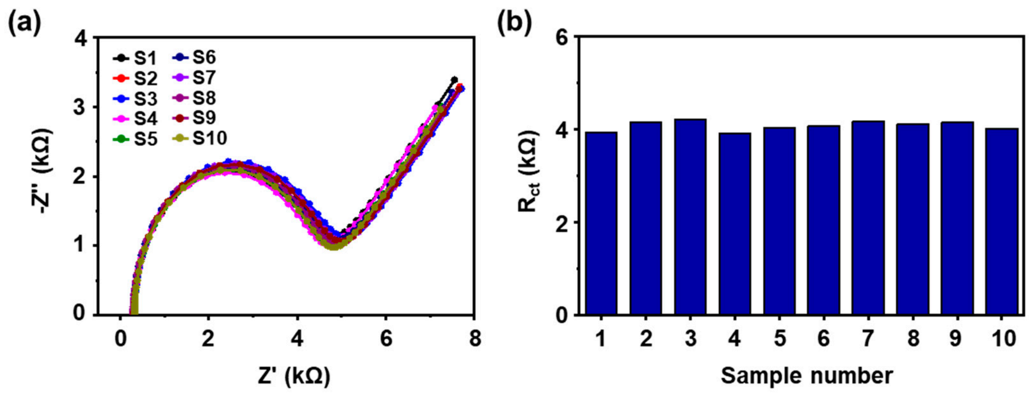

Figure S3. The Nyquist plot (a) and corresponding R_{ct} value (b) after pretreatment of the customized SPCE; the pretreatment was performed using CV under -0.65–1.05 V potential range, 50 mV s^{-1} scan rate, 10 cycles in 0.5 M H_2SO_4 solution.

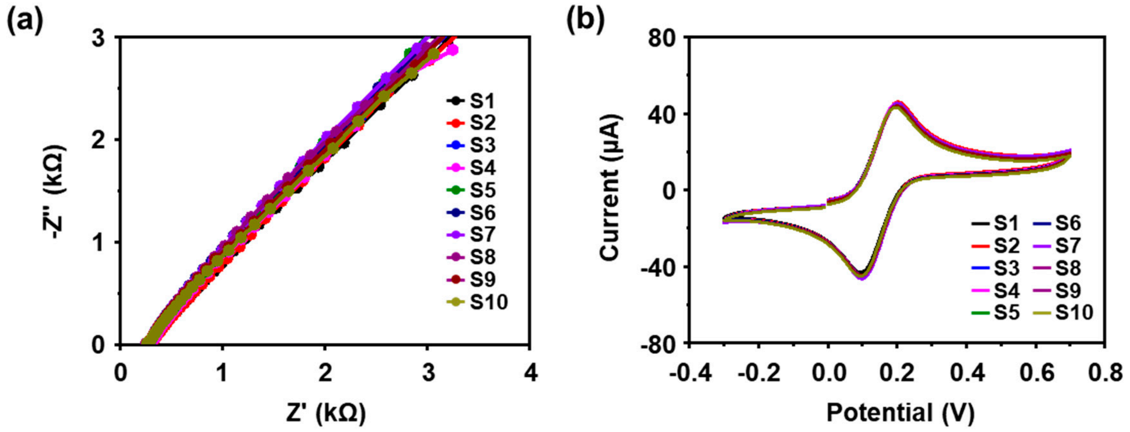

Figure S4. The Nyquist plot (a) and CV voltammogram (b) after electrochemical deposition of PEDOT on SPCE surface; each PEDOT film was electropolymerized using CA under a fixed potential (10 s at 0.9 V) in 0.1 M PBS buffer.

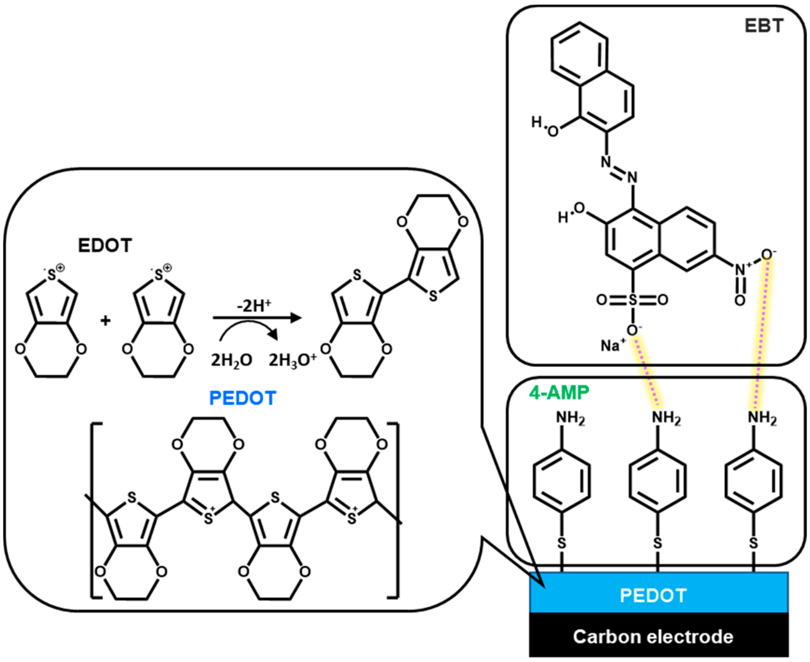

Figure S5. Schematic diagram of a MIP-based biosensor fabricated on a SPCE: sequentially composed of a PEDOT film, a 4-AMP monolayer, and a PEBT film.

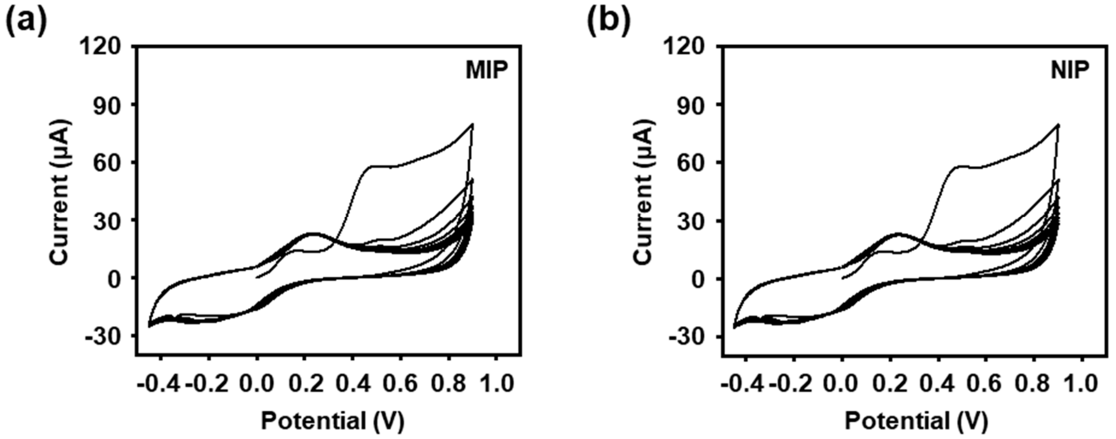

Figure S6. Consecutive CV curves obtained during the electrodeposition of EBT on the 4-AMP/PEDOT/SPCE (a) with and (b) without IL-1β templates.

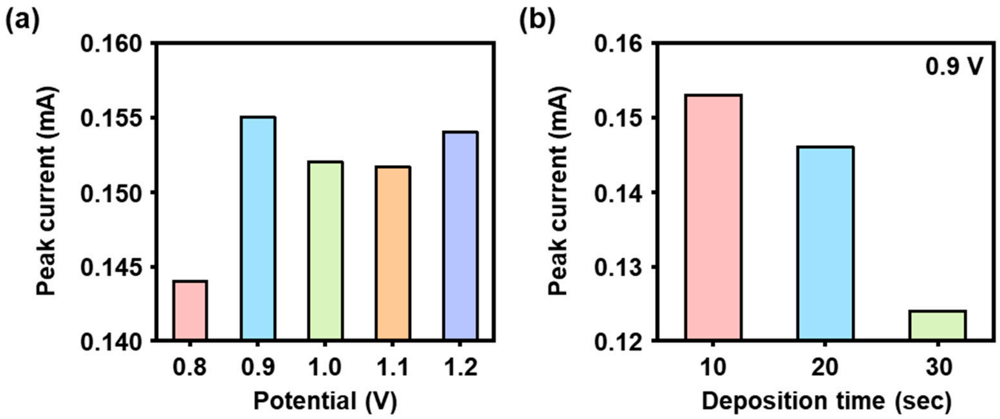

Figure S7. Electrochemical response of PEDOT film on SPCE surface electropolymerized by adjusting deposition voltage (a) and time (b); each peak current was obtained using CV under the parametric condition of a -0.3–0.1 V potential range and 100 mV s^{-1} scan rate in PBS solution (pH = 7.4) containing 5 mM of $[Fe(CN)6]3^{-}/4^{-}$.

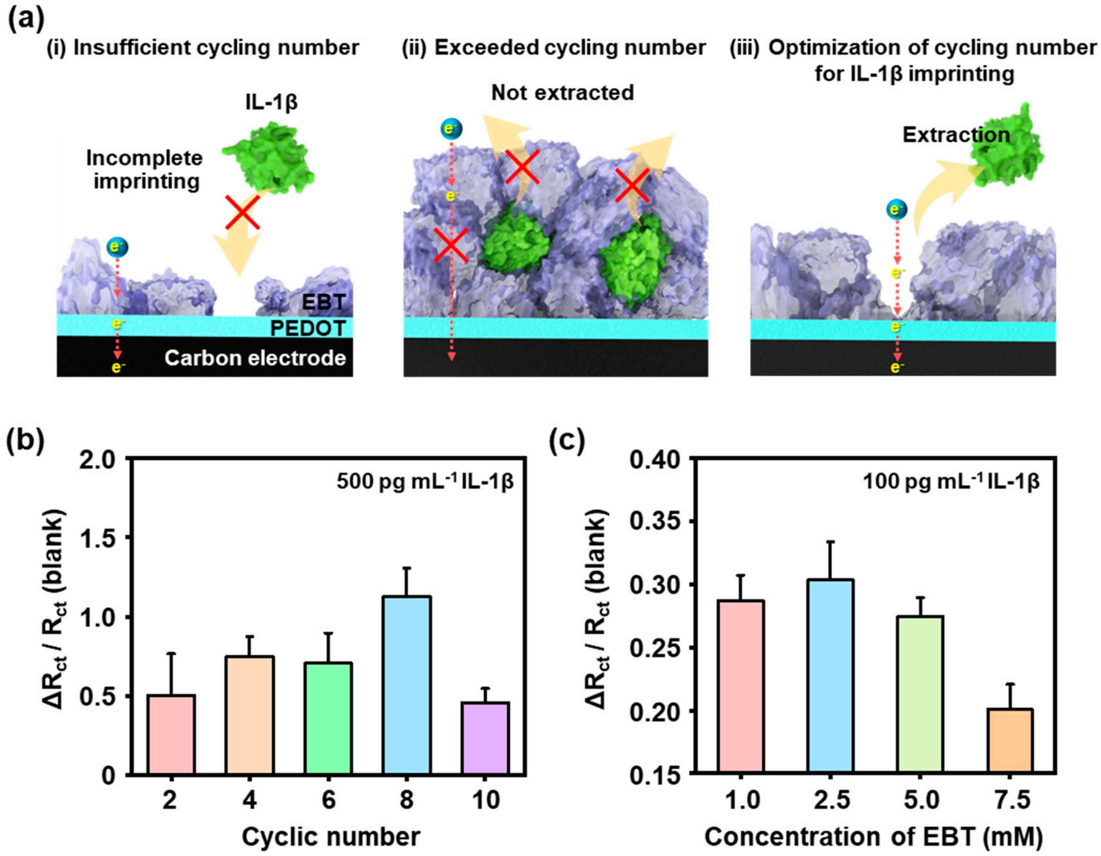

Figure S8. (a) Schematic illustration representing the correlation between the thickness of the MIP matrix and the reliability of the MIP-based biosensor: (i) incomplete imprinting, (ii) entrapped IL-1β templates by a thick PEBT film, and (iii) the formation of the optimized MIP thickness adjustment for IL-1β. The influence of the number of polymerization cycles (b) and the monomer/template concentration ratios (c) to survey the impedimetric response of the PEBT-based MIP sensor to IL-1β.

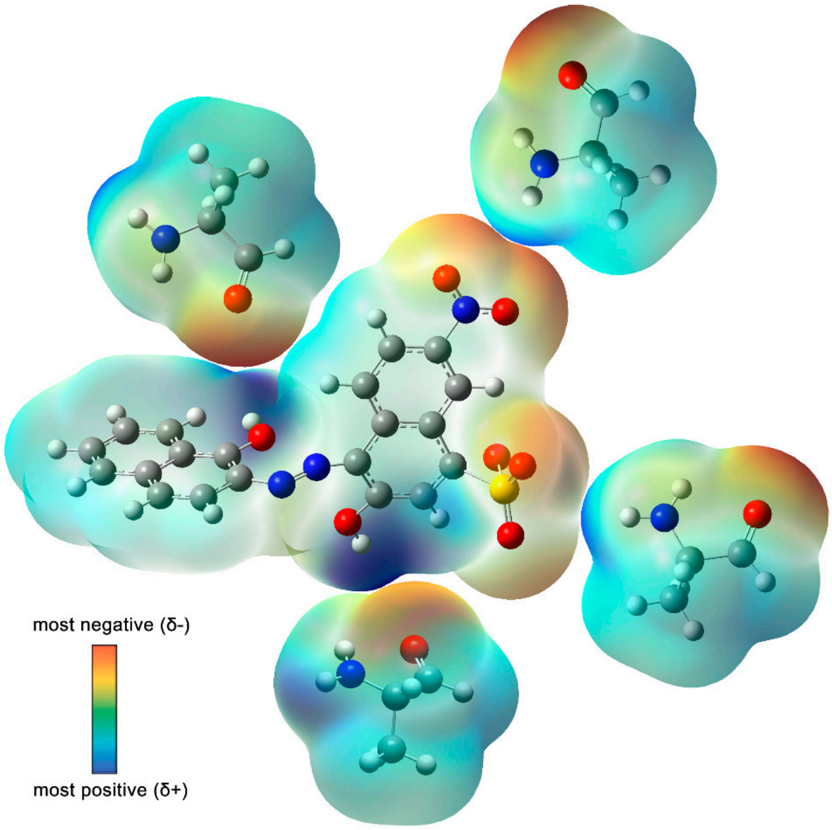

Figure S9. The docking structures (i.e., before hydrogen bond formation) of the amino acid (alanine) tem-plates with EBT monomer, describing the sites of dipole (δ−)–dipole (δ+) interactions. The heatmap displays the electrostatic potential mapping on the isovalue surface at a total electron density of 0.0004 using the self-consistent field method. Because the continuous carbon skeleton of the amino acid is connected to the terminal group, a neutral molecular orbital is not formed for a single sequence. Therefore, in the simulation, the terminals of the amino acid chain were hy-drogenated to assume a neutral structure.

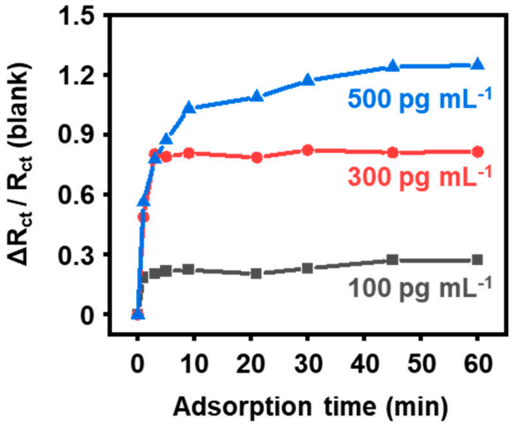

Figure S10. The normalized $\Delta R_{ct}/R_{ct(blank)}$ values as a function of the incubation time were derived from Nyquist plots obtained through dynamic EIS response for the MIP films. $R_{ct(blank)}$ signifies the measured R_{ct} value of the MIP film before incubation in the specific concentration of IL-1β standard solution.

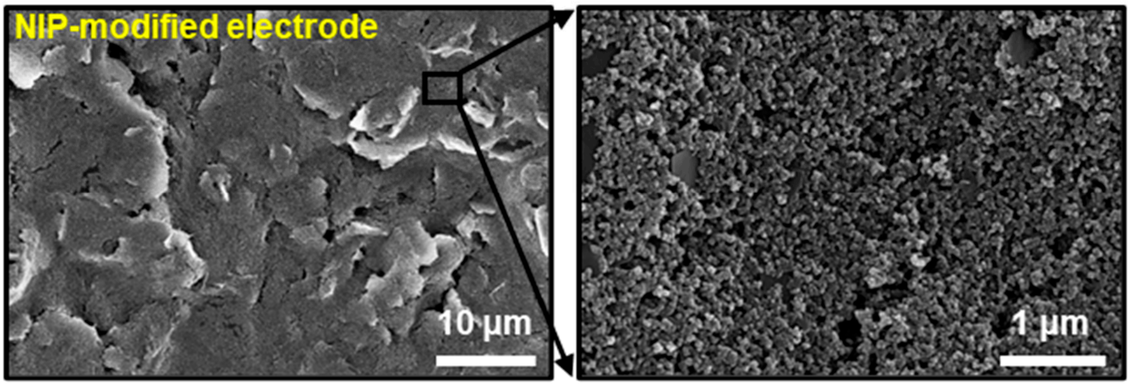

Figure S11. SEM images showing the surface morphology of NIP films fabricated on 4-AMP/PEDOT/SPCE.

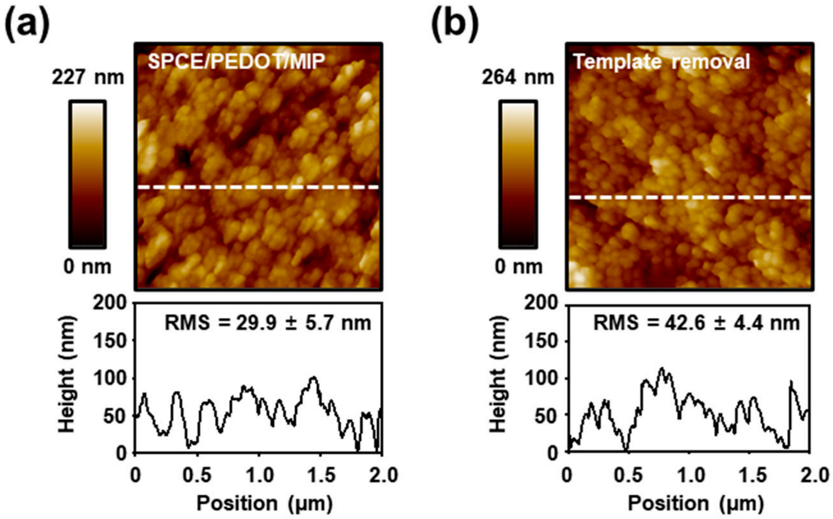

Figure S12. AFM images for topological features of the MIP film (a) before and (b) after the extraction of IL-1β templates.

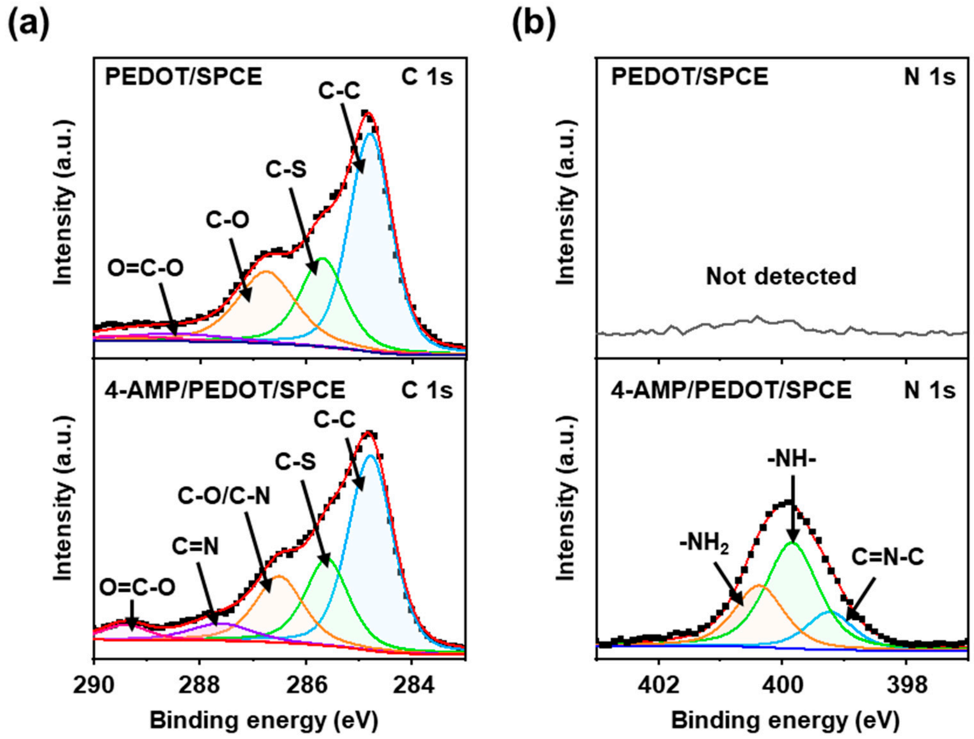

Figure S13. High-resolution XPS spectra of (a) PEDOT/SPCE and (b) 4-AMP/PEDOT/SPCE before the electrodeposition of EBT with IL-1β template.

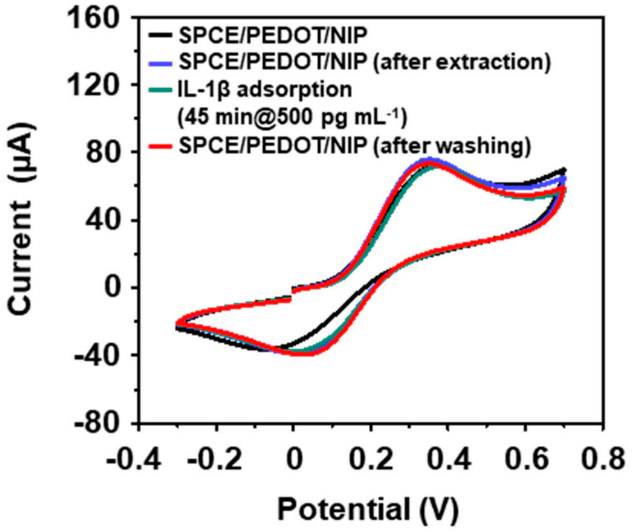

Figure S14. Electrochemical behaviors of NIP/4-AMP/PEDOT/SPCE according to the stepwise-fabrication process measured using CV in 0.01 M PBS (pH 7.2) containing the 5 mM $[Fe(CN)_6]^{3-/4-}$ redox probe.
